# Periostin^+^ myeloid cells improved long bone regeneration in a mechanosensitive manner

**DOI:** 10.1038/s41413-024-00361-5

**Published:** 2024-10-15

**Authors:** Ziyan Wang, Minmin Lin, Yonghao Pan, Yang Liu, Chengyu Yang, Jianqun Wu, Yan Wang, Bingtong Yan, Jingjing Zhou, Rouxi Chen, Chao Liu

**Affiliations:** 1 Department of Biomedical Engineering, Southern University of Science and Technology, Shenzhen, Guangdong, 518055 China; 2 Department of Materials Science and Engineering, Southern University of Science and Technology, Shenzhen, Guangdong, 518055 China; 3 Guangdong Provincial Key Laboratory of Advanced Biomaterials, Southern University of Science and Technology, Shenzhen, Guangdong, 518055 China

**Keywords:** Bone, Bone quality and biomechanics

## Abstract

Myeloid cells are pivotal in the inflammatory and remodeling phases of fracture repair. Here, we investigate the effect of periostin expressed by myeloid cells on bone regeneration in a monocortical tibial defect (MTD) model. In this study, we show that periostin is expressed by periosteal myeloid cells, primarily the M2 macrophages during bone regeneration. Knockout of periostin in myeloid cells reduces cortical bone thickness, disrupts trabecular bone connectivity, impairs repair impairment, and hinders M2 macrophage polarization. Mechanical stimulation is a regulator of periostin in macrophages. By activating transforming growth factor-β (TGF-β), it increases periostin expression in macrophages and induces M2 polarization. This mechanosensitive effect also reverses the delayed bone repair induced by periostin deficiency in myeloid cells by strengthening the angiogenesis-osteogenesis coupling. In addition, transplantation of mechanically conditioned macrophages into the periosteum over a bone defect results in substantially enhanced repair, confirming the critical role of macrophage-secreted periostin in bone repair. In summary, our findings suggest that mechanical stimulation regulates periostin expression and promotes M2 macrophage polarization, highlighting the potential of mechanically conditioned macrophages as a therapeutic strategy for enhancing bone repair.

## Introduction

Bone fracture repair is a complex and highly controlled process, encompassing distinct and interconnected stages of inflammation, repair, and remodeling. The inflammatory and regenerative phases during fracture repair involve the participation of macrophages, which originate from tissue-resident macrophages or recruited monocytes.^1,2^ Both the numbers and subtypes of macrophages are the regulatory factors in the process of bone regeneration. Inadequate numbers of F4/80^+^ macrophages induce smaller and more fibrous fracture calluses, leading to delayed bone repair.^3,4^ The subtypes of macrophages are influenced by a range of cytokines and chemokines, and their polarization states are dynamic in response to local microenvironmental changes.^5^ M1 and M2 macrophages are recruited early during fracture repair and jointly regulate the inflammatory response and remodeling phases of bone repair.^6^ M1 macrophages amplify and attempt to clear pathogens or foreign substances from the damaged site by producing pro-inflammatory cytokines such as tumor necrosis factor-α (TNF-α), inducible nitric oxide synthase (iNOS), and reactive oxygen species (ROS).^7,8^ They also contribute to enhanced bone resorption through the secretion of pro-inflammatory cytokines and act as a reservoir for osteoclasts.^9–11^ However, prolonged activation of M1 macrophages may disrupt the M1/M2 balance, leading to tissue damage, chronic inflammation, and delayed tissue repair. M2 macrophages and their secretion of potential osteogenic cytokines, such as vascular endothelial growth factor (VEGF), bone morphogenetic protein 2 (BMP-2), and TGF-β, play a crucial role in stimulating preosteoblast activation, differentiation, and bone mineralization.^6,12,13^ The contribution of macrophage subtype is essential in bone healing, while the precise molecular mechanisms governing macrophage regulation of bone defect repair are not fully understood.

Periostin is a 90 kD extracellular matrix protein initially identified as osteoblast-specific factor 2 in the mouse osteoblastic cell line MC3T3-E1.^14^ In the skeletal system, periostin participates in various phases of bone repair, including the recruitment of osteoprogenitors to the callus during the initial inflammatory and angiogenic stages, as well as the subsequent regulation of osteoblastic differentiation and bone formation.^15^ Rapid upregulation of periostin occurs in the process of fracture healing, particularly in proliferating mesenchymal stem cells (MSCs) and osteoblasts.^16^ Periostin facilitates the differentiation and osteogenesis of periodontal ligament MSCs,^17,18^ and inhibits osteoclast differentiation.^19^ Conversely, the absence of periostin leads to the defective attachment of osteoblasts to the bone matrix, affecting their maturation into osteocytes.^20^ In addition to osteogenic cells, TRAP^+^ macrophages secrete periostin to attract angiogenic and osteogenic progenitors to the periosteum, resulting in the formation of type H vessels during bone defect repair.^21^ Periostin acts as a signaling molecule that binds to integrin receptors, promotes the recruitment of macrophages, inhibits the expression of pro-inflammatory factors, and contributes to polarization towards the M2 subtype.^22,23^ Previous studies have primarily focused on the role of periostin in macrophage recruitment and polarization, while its functions as a macrophage-secreted factor that regulates osteogenic cells remain poorly understood.

The skeletal system is sensitive to mechanical loading, which significantly impacts bone development and repair by directly affecting osteogenic cells, vascular endothelial cells, and macrophages. Dynamic mechanical loading applied during the matrix deposition phase of bone repair promotes bone formation by increasing the proliferation and differentiation of Prrx1^+^ osteogenic progenitors.^24,25^ Mechanical loading improves skeletal blood perfusion, angiogenic activity, and the formation of type H vessels by activating YAP in vascular endothelial cells.^26–28^ Furthermore, macrophages are present in almost all peripheral tissues and are influenced by various mechanical environments, thereby altering their function.^29^ Macrophages exhibit diverse cellular responses when subject to mechanical forces of various types and magnitudes. Centrifugal force stimulates macrophage differentiation into osteoclasts.^30^ Low shear stress induces M1 macrophage polarization, while high shear stress induces M2 macrophage polarization.^31,32^ Mechanical loading can enhance bone repair by upregulating macrophage differentiation into the M2 subtype and stimulating the secretion of pro-osteogenic signals.^33,34^ Macrophages possess the ability to perceive distinct mechanical stimuli and adapt their functions accordingly, suggesting the possibility of regulating bone modeling and remodeling by appropriately manipulating mechanically conditioned macrophages.

In this study, we employed a stable monocortical tibial defect (MTD) model in mice, a widely used in vivo model for intramembranous bone repair.^25,35^ We utilized *LysM-Cre; tdTomato* mice to investigate the localization of myeloid cells during bone repair. During bone defect repair, periostin was continuously expressed in M2 macrophages and played a critical role in facilitating the polarization of M2 macrophages. To investigate the mechano-regulation of periostin expression in macrophages, we conditionally knocked out periostin in myeloid cells using *LysM-Cre; POSTN*^*−/−*^ mice. As periostin expression was regulated by mechanical stimulation, the impaired bone repair resulting from periostin knockout in myeloid cells could be restored by the application of mechanical loading, which compensated for this deficiency by affecting angiogenesis-osteogenesis coupling. These findings suggested that mechanically conditioned macrophages were a potential therapeutic approach for bone healing.

## Results

### Periosteal myeloid cells participated in bone defect repair

**Fig. 1:**
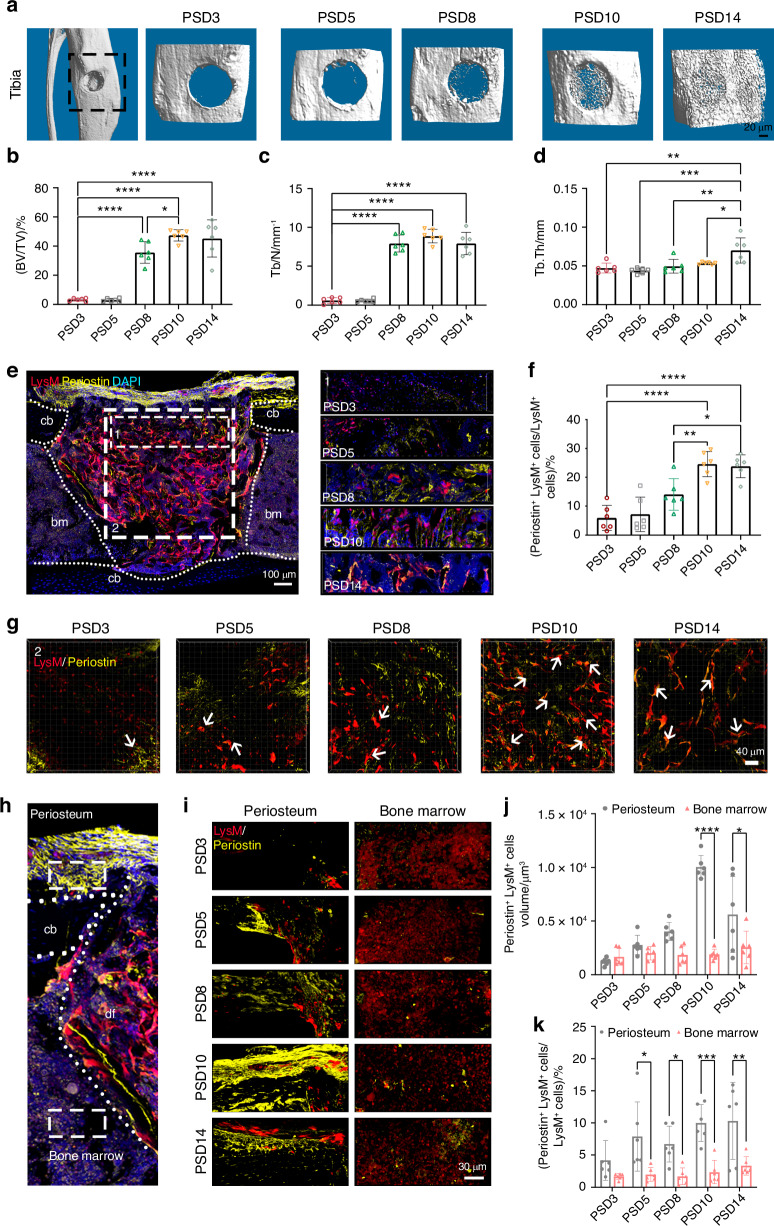
Myeloid cells express periostin during bone regeneration. **a** Three-dimensional (3D) Micro-CT images of new bone accrual isolated from tdTomato mice. Micro-CT analysis of new bone from **b** bone BV/TV, **c** Tb. N, and **d** Tb.Th (*n* = 6). **e** Colocalization of LysM^+^ cells and periostin at the defect site in tdTomato mice on PSD 3, 5, 8, 10, and 14. **f** Quantification of the ratio of periostin^+^ LysM^+^ cells in total LysM^+^ cells (*n* = 6). **g** Colocalization of LysM and periostin (white arrow) in throughout the defect site. **h** Schematic diagram of the regions of LysM^+^ cells origin during bone defect repair in tdTomato mice. **i** Colocalization of LysM and periostin in the region of periosteum and bone marrow. **j** Quantification of the volume of periostin^+^ LysM^+^ cells in the region of periosteum and bone marrow (*n* = 6). **k** Quantification the ratio of periostin^+^ LysM^+^ cells in LysM^+^ cells in the region of periosteum and bone marrow (*n* = 6). cb cortical bone, df defect site, PSD postsurgical day. *P < 0.05; **P < 0.01; ***P < 0.001; ****P < 0.000 1. Ordinary one-way ANOVA. Data were mean ± SD

The immune system interacted with the skeletal system during bone defect repair. Myeloid cells could express periostin in the defect site during the bone repair process.^36^ Therefore, we postulated that periostin could be involved in the regulation of myeloid cells on bone repair. We constructed a myeloid cell lineage-tracing mouse line *LysM-Cre; tdTomato* (tdTomato), and monitored the expression of periostin during bone repair. Almost no new bone tissue was observed in the defect site on post surgical day (PSD) 3 and 5. The new bone began to accumulate on PSD8 and filled the entire defect site on PSD 10. Damaged areas of cortical bone were completely replaced by new bone on PSD 14 (Fig. 1a), as indicated by increased bone volume (BV/TV, Fig. 1b), trabecular number (Tb. N, Fig. 1c) and trabecular thickness (Tb. Th, Fig. 1d). The increased bone stiffness as observed by the reduction in strain concentration at the defect site served as further evidence for the bone repair (Fig. S1a, b). These results demonstrated that the MTD model mimicked the distinct stages of the bone regeneration process within 14 days, allowing the observation of myeloid cells behaviors during this period. Next, we examined the expression of periostin in myeloid cells in the bone defect. Large numbers of myeloid cells expressed periostin from PSD 3 to PSD 14, with higher numbers on PSD 10 and 14, as observed by colocalization and spatial analysis of myeloid cells and periostin (Figs. 1e, f, S1c, d). Similar results were obtained by colocalization and spatial analysis of myeloid cells and periostin throughout the defect site (Figs. 1g, S1e–g). These results suggested that myeloid cells participated in the repair process of bone defects by secreting periostin, especially in the later stage of repair.

To further investigate the differences in the contribution of skeletal location, myeloid cells were categorized into periosteum-localized or bone marrow-localized (Fig. 1h), and their expression of periostin was examined on PSD 3, 5, 8, 10, and 14. myeloid cells located in both the periosteum and bone marrow secreted periostin during bone repair (Fig. 1i). Periostin expression in periosteum-localized myeloid cells increased continuously, whereas in bone marrow-localized myeloid cells, periostin remained at a nearly constant low level throughout the repair process (Fig. 1j, k). These data demonstrated that periosteum-located myeloid cells contributed more to periostin expression than bone marrow-located myeloid cells during bone repair.

### Majority of periostin^+^ macrophages were M2 polarized in areas of bone formation

**Fig. 2:**
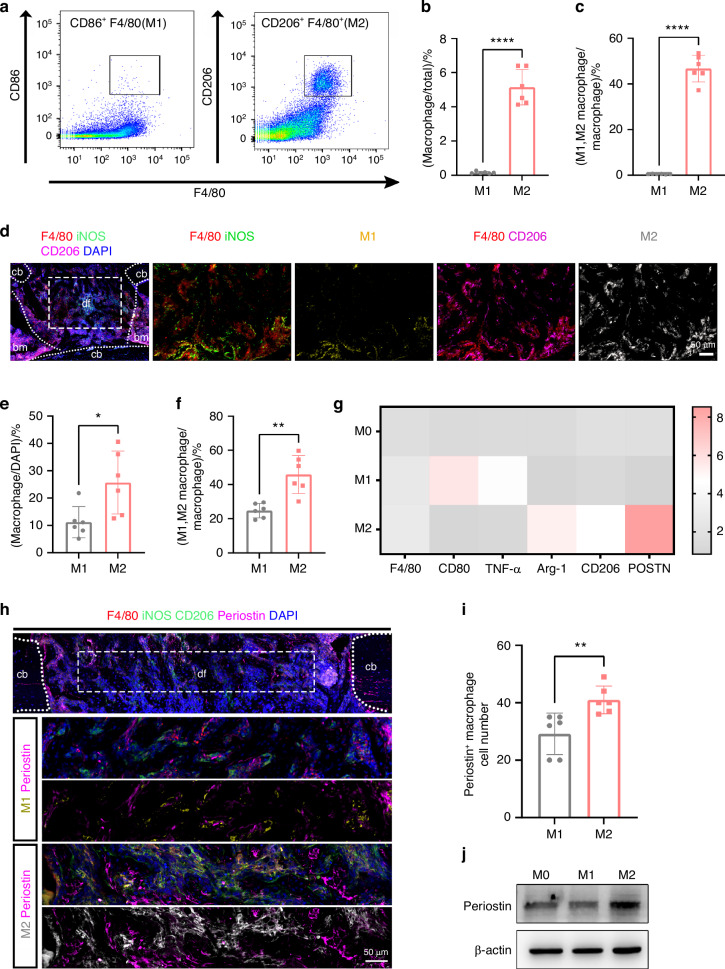
Periostin was primarily expressed by M2 macrophages in the bone defect. **a** Flow cytometry analysis of F4/80^+^ CD86^+^ macrophages (M1 macrophages) and F4/80^+^ CD86^+^ macrophages (M2 macrophages) isolated from the defect site in WT mice. Quantification of FACS results for the proportion of M1 or M2 macrophages in **b** total cells or **c** F4/80^+^ macrophages (*n* = 6). **d** The number of F4/80^+^ iNOS^+^ macrophages (M1 macrophages) and F4/80^+^ CD86^+^ macrophages in WT mice on PSD 10. Quantification of immunofluorescence analysis results for the proportion of M1 or M2 macrophages in **e** total cells and **f** macrophages (*n* = 6). **g** Quantitative of real-time PCR (RT-PCR) analyses of the expression of periostin, and M1 or M2 macrophages marker isolated from WT mice in M0, M1, and M2 macrophages (*n* = 3). **h** The number of periostin^+^ M1 or periostin^+^ M2 macrophages in the defect site of WT mice on PSD 10. **i** Quantification of immunofluorescence analysis results for the number of periostin^+^ M1 or periostin^+^ M2 macrophages (*n* = 6). **j** Western blot analysis of the expression of periostin in M0, M1, and M2 macrophages from WT mice. cb cortical bone, df defect site, bm bone marrow (*n* = 3). *P < 0.05; **P < 0.01; ****P < 0.000 1. Student’s *t* test. Data were mean ± SD

Both M1 and M2 macrophages were present in the process of bone repair.^37^ To uncover the state of macrophage polarization during bone regeneration, we quantified the numbers of M1 and M2 macrophages in the bone defect site on PSD 10. Fluorescence-activated cell sorting (FACS) analysis revealed a significantly higher number of F4/80^+^ CD206^+^ macrophages (M2 marker) compared to F4/80^+^ CD86^+^ macrophages (M1 marker) in both sorted total cells and isolated F4/80^+^ macrophages from the defect site of wild type (WT) mice (Figs. 2a–c, S2a). Moreover, by co-staining F4/80, iNOS, and CD206, the numbers of M1 macrophages (F4/80^+^ iNOS^+^) and M2 macrophages (F4/80^+^ CD206^+^) were calculated simultaneously (Fig. 2d). Higher numbers of M2 macrophages were observed at the whole defect site compared to M1 macrophages (Fig. 2e, f). These data showed that M2 macrophage was the key subtype in the defect site during bone repair. Next, we evaluated the expression of periostin in different subtypes of macrophages. The polarization of M0 to M1 or M2 macrophages was verified by assessing the mRNA expression of CD80, TNF-α (M1 macrophage marker), CD206 and Arg1 (M2 macrophage marker), and their cell morphology (Figs. 2g, S2b). In vitro, higher levels of periostin mRNA expression were identified within M2 macrophages, while lower levels were found in M1 macrophages (Fig. 2g). Periostin protein level was also the highest in M2 macrophages, and lowest in M1 macrophages (Figs. 2j, S2c). In vivo, the higher number of M2 macrophages co-localized with periostin compared with M1 macrophages (Fig. 2h, i). Although both M1 and M2 macrophages can express periostin, M2 macrophages were the predominant subtype in bone defects on PSD10 and expressed more periostin. Thus, M2 macrophages played a key role in promoting bone regeneration.

### Periostin deficiency in myeloid cells led to reduced bone quantity

**Fig. 3:**
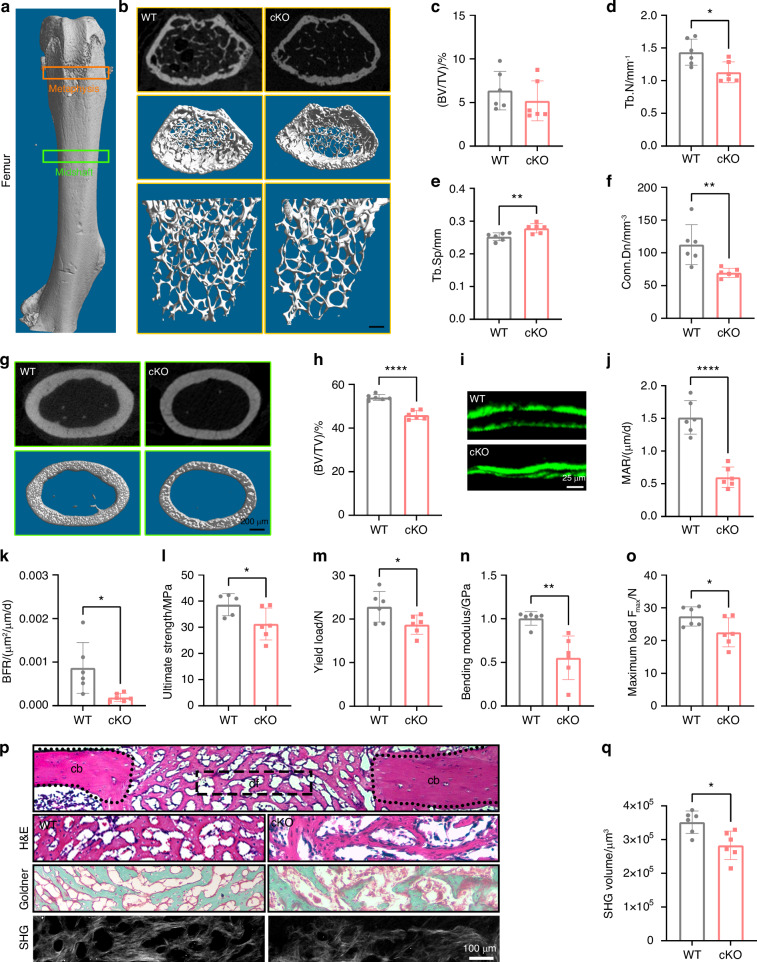
Knockout of periostin in myeloid cells resulted in reduced bone formation. **a** Schematic diagram of selected position of trabecular and cortical bone in the mouse femur metaphysis and diaphysis. **b** 2D and 3D Micro-CT images of trabecular bone of WT mice and cKO mice. Quantitative parameters of Micro-CT analysis of trabecular bone, including **c** BV/TV, **d** Tb. N, **e** Tb. Th, and **f** Conn.Dn (*n* = 6). **g** 2D and 3D Micro-CT images of cortical bone of WT mice and cKO mice. **h** Quantitative of Micro-CT parameter cortical bone BV/TV (*n* = 6). **i** Representative images of dual calcein labeling of femurs from WT mice and cKO mice. Quantification of **j** MAR, and **k** BFR of periosteal bone of the diaphysis of femur (*n* = 6). Quantitative of four point-bending of mouse femur from WT mice and cKO mice, including **l** ultimate strength, **m** yield load, **n** bending modulus, and **o** the maximum load (*n* = 6). **p** Histologic imaging of the healing bone defect by H&E staining, Goldner staining, and two-photon collagen imaging in WT mice and cKO mice. **q** Quantitative of the volume of SHG in WT mice and cKO mice (*n* = 6). cb cortical bone, df defect site, SHG second harmonic generation. *P < 0.05; **P < 0.01; ****P < 0.000 1. Student’s *t* test. Data were mean ± SD

To investigate whether periostin expressed by myeloid cells promotes bone formation, we generated a myeloid cell-specific periostin deletion mouse model (cKO mice). Western blot and confocal imaging confirmed the deletion of periostin in macrophages of cKO mice (Fig. S3a, b). The body size of WT and KO mice was similar (Fig. S4a), and the body weight during development was consistent between these two subtypes (Fig. S4b). However, micro-CT results showed lower bone density in cKO mice, including voids in vertebrae and poor skull ossification (Fig. S4c, d). To further determine the function of myeloid cells-expressed periostin in the skeletal system, we measured the bone quality of cortical bone and trabecular bone in WT mice and cKO mice (Fig. 3a). As shown in Fig. 3b–f, knockout of periostin in myeloid cells reduced trabecular bone thickness (Tb. Th), trabecular number (Tb. N) and connectivity density (Conn.Dn), with no changes in trabecular bone volume per tissue volume (BV/TV). Further analysis of cortical bone in cKO mice displayed a decline in cortical bone thickness, accompanied by lower bone volume (BV/TV), cross-sectional bone area per tissue area (B.Ar/T.Ar), and average principal moment of inertia (max) (Av. MMI (max)) (Figs. 3g, h, S4e, f). Moreover, dynamic histomorphometry showed significantly reduced bone mineral apposition rate (MAR) and bone formation rate (BFR) in cKO mice (Fig. 3i–k). Four-point bending confirmed decreased bone biomechanical properties of cKO mice in ultimate strength (Fig. 3l), yield load (Fig. 3m), bending modulus (Fig. 3n), and the maximum load (Fig. 3o). Together, periostin cKO mice had abnormal morphology and impaired bone formation. Moreover, we evaluated the effect of periostin cKO in myeloid cells on bone regeneration. Periostin cKO mice showed impaired bone defect repair marked by reduced collagen formation, lower new bone accrual, and less mineralized bone on PSD 10 (Fig. 3p, q). Delayed bone healing and worse bone mechanical properties were also observed in the mouse osteotomy model (Fig. S4g, h). These results demonstrated that the knockout of periostin in myeloid cells had inhibitory effects on bone modeling and repair.

### Inactivation of periostin in myeloid cells caused impaired M2 polarization

**Fig. 4:**
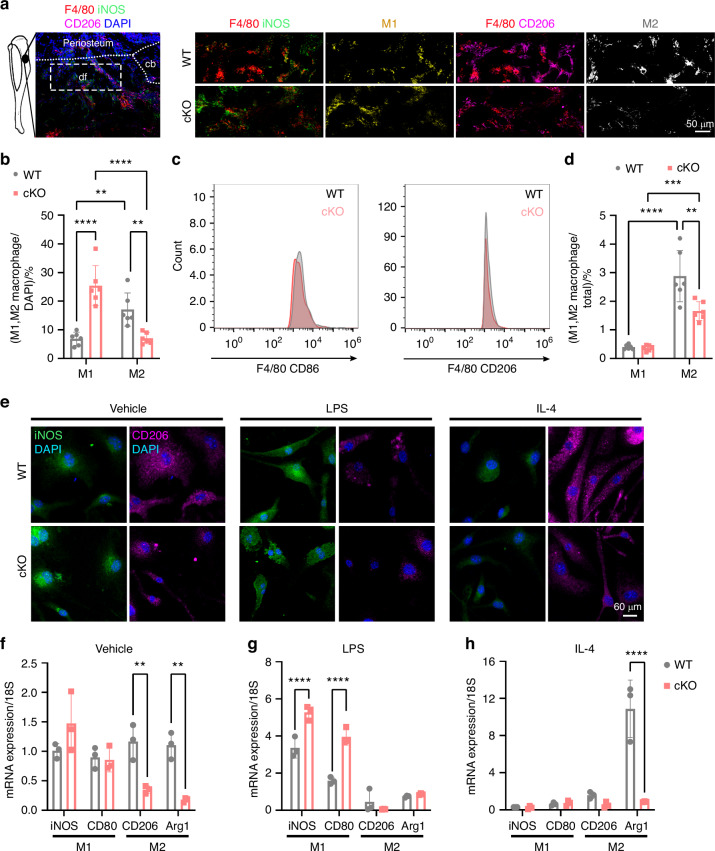
Knockout of periostin in myeloid cells inhibited M2 polarization. **a** The number of M1 or M2 macrophages in the defect site of WT or cKO mice on PSD 10. **b** Quantification of immunofluorescence staining of M1 or M2 macrophages out of total macrophages (*n* = 6). **c** FACS analysis of M1 or M2 macrophages isolated from the defect site in WT or cKO mice. **d** Quantification of FACS analysis of the proportion of M1 or M2 macrophages in total cells (*n* = 6). **e** Expression of iNOS and CD206 in BMDMs of WT or cKO mice under in vehicle-, LPS- and IL-4- treatment (*n* = 3). Quantitative RT-PCR analyses of the expression of M1 and M2 BMDMs isolated from WT or cKO mice in **f** vehicle-, **g** LPS- and **h** IL-4- treatment (*n* = 3). cb cortical bone, df defect site. **P < 0.01; ***P < 0.001; ****P < 0.000 1. Ordinary two-way ANOVA. Data were mean ± SD

To elucidate the mechanisms underlying the lower regenerative potential of macrophages in cKO mice compared to WT mice during bone healing, we analyzed macrophage behavior in the bone defect and bone marrow-derived macrophages (BMDMs). Immunofluorescence analysis revealed a higher number of M2 macrophages compared to M1 macrophages in WT mice. Conversely, cKO mice displayed a decrease in M2 polarized cells and an increase in M1 polarized cells at PSD 10 (Fig. 4a, b). Fewer M2 macrophages were present in sorted F4/80^+^ cells isolated from the bone defect site of cKO mice, but no difference in the number of M1 macrophages (Fig. 4c, d). FACS sorting analysis revealed no significant difference in the interconversion between M1 and M2 macrophages in WT and cKO mice (Fig. S5a–c). Moreover, we analyzed cell behaviors of BMDMs isolated from WT or cKO mice. Periostin deletion in macrophages did not compromise their proliferation, as shown by similar numbers of F4/80^+^ macrophages at the defect site in vivo, and similar numbers of Ki67^+^ macrophages in vitro (Fig. S5d–g). Cell scratch analysis exhibited no differences in cell migration of BMDMs from WT or cKO mice, which failed to explain the low regenerative ability of the periostin-deactivated myeloid cells (Fig. S5h, i). These results demonstrated that knockout of periostin in myeloid cells did not affect macrophages proliferation and migration, thus we hypothesized the impaired potential of bone healing was due to the regulation of macrophage polarization and differentiation. Immunofluorescence and histological staining results showed that knocking out of periostin in myeloid cells increased the number of TRAP^+^ osteoclasts in bone defects (Fig. S5j). In vitro, the results demonstrated that after RANKL treatment, bone marrow macrophages from cKO mice can expressed more TRAP^+^ cells compared to WT mice (Fig. S5k). These results indicated that knocking out periostin in macrophages induced osteoclast differentiation. Furthermore, immunofluorescence analysis confirmed the reduced ability of M2 polarization in the absence of periostin (Fig. 4e). M1 (iNOS, CD80) and M2 (CD206, Arg1) macrophage markers in BMDMs from WT or cKO mice were measured by RT-PCR. BMDMs from cKO mice had lower expression of M2-specific markers compared to WT (Fig. 4f). Treatment with macrophage activator Lipopolysaccharide (LPS) induced higher expression of M1-specific markers in cKO BMDMs compared to WT, but had no effect on M2-specific markers (Fig. 4g). Treatment with IL-4 only affected Arg1 expression, which indicated that loss of periostin in macrophages affected the polarization to M1 and M2 subtype, but not interconversion between M1 and M2 macrophages (Fig. 4h). Together, periostin in macrophages was a key effector in M2 polarization, without regulating macrophages proliferation and migration.

### Mechanical loading increased M2 macrophage numbers and periostin expression in bone defect

**Fig. 5:**
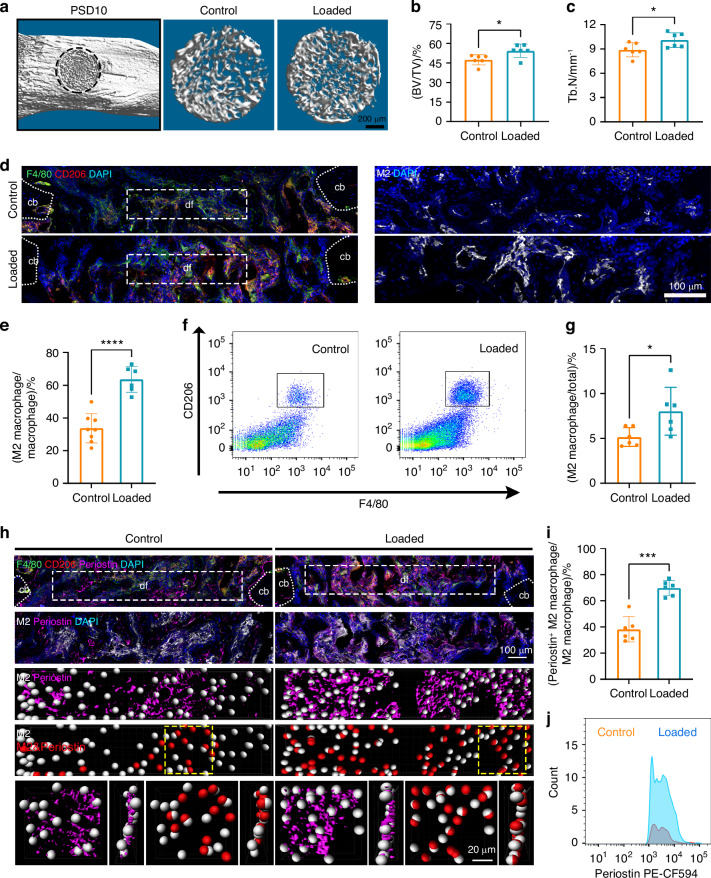
Macrophages polarized towards M2 and expressed periostin in response to mechanical loading. **a** 3D Micro-CT images of new bone accrual isolated from WT mice on PSD 10 after MTD surgery in control and loaded tibia. Quantitative parameters of Micro-CT analysis of new bone accrual including **b** BV/TV and **c** Tb.N (*n* = 6). **d** The number of M2 macrophages in control and loaded tibia of WT mice on PSD 10. **e** Quantification of immunofluorescence analysis of the proportion of M2 macrophages in total macrophages (*n* = 6). **f** Flow cytometry analysis of M2 macrophages isolated from defect site in control and loaded tibia. **g** Quantification of FACS analysis of the proportion of M2 macrophages in total cells (*n* = 6). **h** The number of periostin^+^ M2 macrophages in control and loaded tibia of WT mice on PSD 10. **i** Quantification of the proportion of periostin^+^ M2 macrophages in total M2 macrophages (*n* = 6). **j** Flow cytometry analyses of periostin^+^ M2 macrophages isolated from defect site in control and loaded tibia. cb cortical bone, df bone defect, PSD postsurgical day. *P < 0.05; ***P < 0.001; ****P < 0.000 1. Student’s *t* test. Data were mean ± SD

To investigate the mechanisms by which mechanical stimulation improved bone regeneration via macrophages, we applied 6 N peak dynamic mechanical force on the mouse tibia. Mechanical loading increased new bone accrual with higher trabecular bone volume and trabecular number in the defect site on PSD 10, and 14 (Figs. 5a–c, S6a–c). The mechanical stiffness improved at the defect site on PSD 10 and 14 after applied loading as shown by reduced strain concentration (Fig. S6d, e). To study the role of mechanical loading in regulating macrophage activation and polarization, we first assessed whether mechanical loading could regulate the number of F4/80^+^ cells. Mechanical loading increased the number of F4/80^+^ macrophages in the defect site during bone regeneration (Fig. S7a, b). Similar results were obtained by FACS analysis of macrophages from the defect site, indicating that loading increased macrophage number (Fig. S7c, d). We next examined the polarization of macrophages in response to mechanical loading during bone defect repair. After exposure to mechanical loading, macrophages polarized to the M2 subtype, which may contribute to the bone repair with anti-inflammatory function (Fig. 5d, e). M2 macrophages were also enriched In sorted F4/80^+^ macrophages isolated from loaded defect site (Figs. 5f, g, S8a, b). Moreover, the numbers of M1 macrophages were simultaneously increased in the defect site (Fig. S8c, d). These data suggested that mechanical loading applied on mouse tibia defect enhanced macrophage number and promoted macrophage polarization towards M1 and M2 subtypes.

We next elucidated how mechanical loading regulated the expression of periostin by macrophages. As majority of periostin-expressing macrophages were M2 polarized (Fig. 2g–j), we focused on periostin expression specifically in M2 macrophages in response to mechanical loading. Within the bone defect region, a higher proportion of periostin^+^ M2 macrophages were present after loading compared with control (Fig. 5h–i). More periostin^+^ M2 macrophages were shown in sorted F4/80^+^ cells isolated from the bone defect site in response to mechanical loading (Fig. 5j). Taken together, these results demonstrated the role of mechanical loading at increasing macrophage number, polarization, and secretion of periostin.

### M2 Macrophage expression of periostin was regulated by mechanical strain

**Fig. 6:**
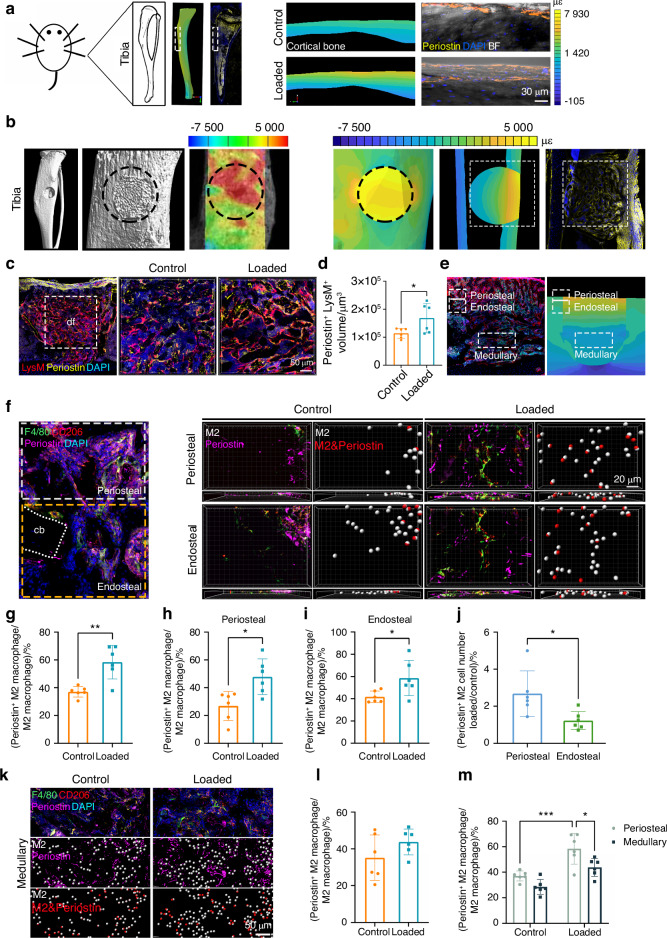
Mechanical stimulation increased periostin expression in macrophages. **a** FEA analysis of the strain distribution in mouse tibia and immunofluorescence staining of periostin expression in the periosteum (*n* = 6). **b** 3D Micro-CT images of schematic diagram of bone defect, DIC and FEA imaging of the distribution of strain, and immunofluorescence imaging of periostin in defect site (*n* = 6). **c** Colocalization of macrophage and periostin in the whole defect site of tdTomato mice. **d** Quantification of the volume of periostin^+^ LysM^+^ in tdTomato mice (*n* = 6). **e** Microstrain distribution in the region of periosteal, and medullary. **f** The number of periostin^+^ M2 macrophages in control or loaded tibia in the region of periosteal and endosteal. **g** Quantification of the proportion of periostin^+^ M2 macrophages in total M2 macrophages in the region of periosteal plus endosteal (*n* = 6). Quantification of the proportion of periostin^+^ M2 macrophages in total M2 macrophages in the region of **h** extramedullary and **i** intramedullary (*n* = 6). **j** Quantification of the ratio of periostin^+^ M2 macrophages changes in periosteal and endosteal region (*n* = 6). **k** The number of periostin^+^ M2 macrophages in control or loaded tibia in the region of medullary. **l** Quantification of the proportion of periostin^+^ M2 macrophages in total M2 macrophages in medullary region (*n* = 6). **m** Quantification of the proportion of periostin^+^ M2 macrophages in total M2 macrophages in control or loaded tibia in the region of periosteal and medullary region (*n* = 6). *P < 0.05; **P < 0.01; ***P < 0.001. Student’s *t* test. Data were mean ± SD

Periostin expression has been shown as mechanosensitive to regulate bone formation.^38^ Under physiological conditions, finite element analysis (FEA) results showed areas of strain concentration in the anteromedial surface of the tibia, accompanied by high expression of periostin (Fig. 6a). The presence of a circular defect resulted in strain concentration at the site, as observed in ex vivo specimens using digital image correlation (DIC). This strain concentration spatially correlated with the expression of periostin (Fig. 6b). Since mechanical loading stimulated periostin expression in all macrophages on PSD 10, we hypothesized that mechanical strain regulated periostin expression in macrophages (Fig. 6c, d). In the bone defect, FEA analysis showed a strain gradient, with a higher strain at the periosteal region, and a lower strain in the medullary region (Fig. 6e). To quantify macrophage number, the tibial defect was divided into two regions: periosteal and endosteal, and the number of periostin^+^ M2 macrophages were calculated (Fig. 6f). Mechanical loading increased the number of periostin^+^ M2 macrophage in the periosteal plus endosteal regions (Fig. 6g). Higher number of M2 macrophages located in both periosteal and endosteal regions expressed periostin after mechanical loading (Fig. 6h, i). However, more periostin^+^ M2 macrophages were detected in the periosteal region compared to the endosteal region after mechanical loading (Fig. 6j). Interestingly, mechanical loading did not alter the expression of periostin^+^ M2 macrophages in the medullary region (Fig. 6k, l). Taken together, a higher number of periostin^+^ M2 macrophages was present in only the periosteal region, not in the medullary region after mechanical loading, which suggested a strain-dependent characteristic of periostin expression in M2 macrophages (Fig. 6m).

### Mechanical loading mitigated the impaired bone formation caused by periostin-deletion

**Fig. 7:**
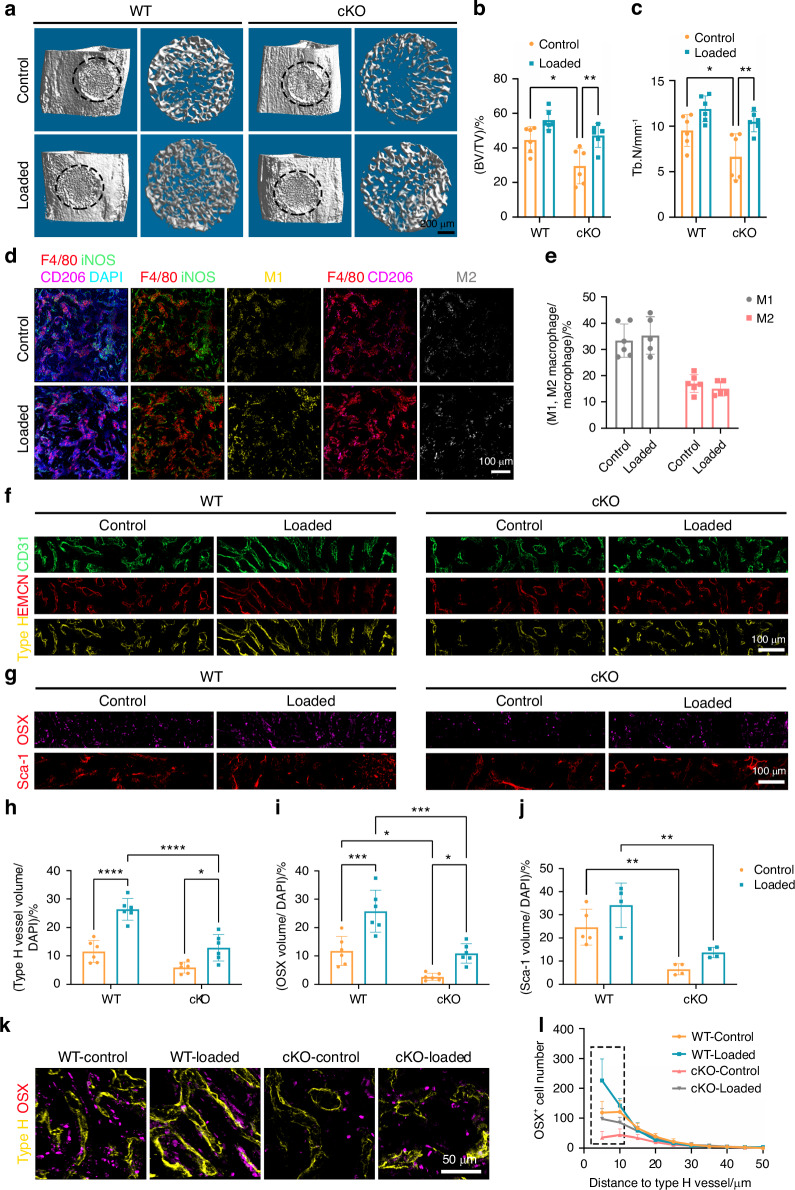
Mechanical loading rescued the impaired osteogenesis and angiogenesis due to periostin knockout. **a** 3D Micro-CT images of new bone accrual isolated from WT or cKO mice on PSD 10 after MTD surgery in control and loaded tibia. b-c Quantitative parameters of Micro-CT analysis of new bone accrual including **b** BV/TV and **c** Tb. N (*n* = 6). **d** The number of M1 and M2 macrophages in control and loaded tibia of cKO mice on PSD 10. **e** Quantification of immunofluorescence analysis of the proportion of M1 and M2 macrophages in total macrophages in control and loaded tibia (*n* = 6). **f** The numbers of CD31^+^ vessels, EMCN^+^ vessels and CD31^+^ EMCN^+^ type H vessels in control and loaded tibia of WT or cKO mice on PSD 10. **g** The number of OSX^+^ cells and Sca-1^+^ cells in control and loaded tibia of WT or cKO mice on PSD 10. **h** Quantification of the volume of type H vessels in control and loaded tibia of WT or cKO mice (*n* = 6). Quantification of the number of osteogenic cells **i** OSX^+^, **j** Sca-1^+^ cells in control and loaded tibia of WT or cKO mice (*n* = 6). **k** OSX^+^ cells and type H vessels in control and loaded tibia of WT mice and cKO mice on PSD 10. **l** Quantification of the OSX^+^ cell numbers in various distances to type H vessels in control and loaded tibia (*n* = 6). *P < 0.05; **P < 0.01; ***P < 0.001; ****P < 0.000 1. Ordinary two-way ANOVA. Data were mean ± SD

Mechanical loading significantly increased new bone formation in the defect of WT mice. To achieve a comparable enhancement from mechanical stimulation in cKO mice, we applied a load that resulted in similar strain levels as experienced by WT mice (Fig. S9a). While cKO mice exhibited lower bone accrual compared to WT mice on PSD 10 (Fig. 7a–c), interestingly, mechanical loading induced higher trabecular bone volume and t number in cKO mice (Fig. 7a–c). The cKO mice had weaker mechanical properties compared to WT, showing significantly higher strains; but mechanical loading reduced strain concentration in cKO mice on PSD 10 (Fig. S9b, c). These data showed that mechanical loading attenuated the poor bone formation and mechanical stiffness caused by periostin deletion in myeloid cells.

**Fig. 8:**
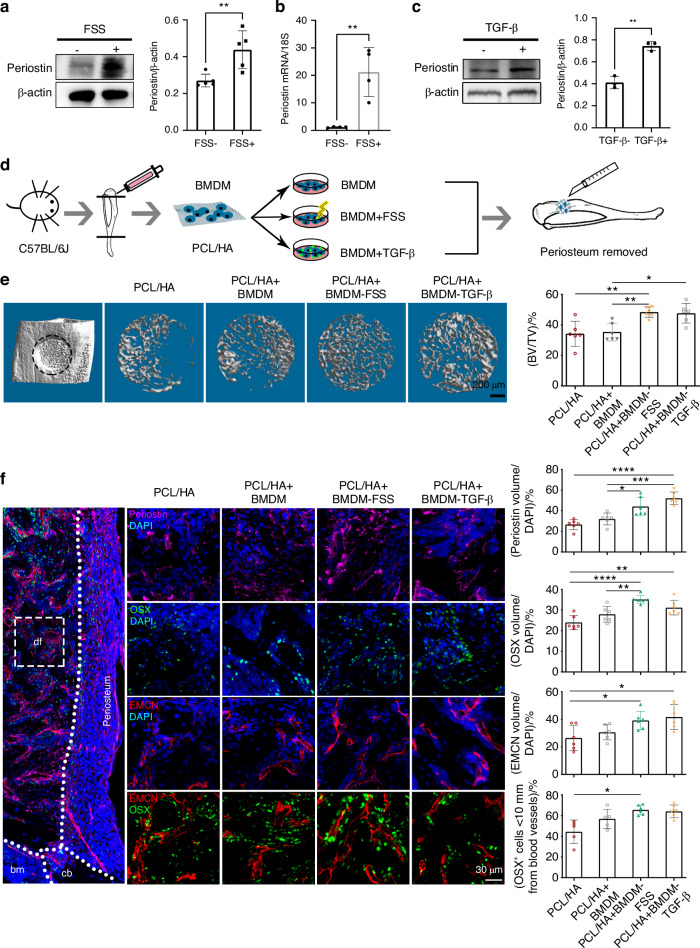
Transplantation of mechanically conditioned macrophages in an artificial periosteum membrane promoted bone regeneration. **a** Western blot analysis and quantitation of the expression of periostin after treated with fluid shear stress in BMDMs (*n* = 5). **b** Quantitative RT-PCR analyses of the expression of periostin after fluid shear stress treatment (*n* = 4). **c** Western blot analysis and quantitation of the expression of periostin after treated with TGF-β in BMDMs (*n* = 3). **d** Experimental design schematic for the isolation of macrophages from C57BL/6 J donors and transplantation at the defect site during bone healing. **e** 3D Micro-CT images and of quantification of the new bone accrual isolated from C57BL/6 J mice on PSD 10 after MTD surgery in PCL/HA, PCL/HA with BMDMs, PCL/HA with FSS-treated BMDMs, PCL/HA with TGF-β-treated BMDMs from C57BL/6 J mice (*n* = 6). **f** Immunofluorescence staining and quantification of the periostin expression, OSX^+^ cells, EMCN^+^ vessels and angiogenesis-osteogenesis coupling on PSD 10 (*n* = 6). cb cortical bone, bm bone marrow, df bone defect. *P < 0.05; **P < 0.01; ***P < 0.001; ****P < 0.000 1. Ordinary one-way ANOVA. Data were mean ± SD

Since mechanical loading did not alter macrophage polarization in periostin knockout mice (Fig. 7d, e), the beneficial effects on bone regeneration in periostin cKO mice likely occurred independently of changes in macrophage subtype. We then assessed the possibility of mechanical stimulation rescuing impaired bone repair by regulating angiogenesis‐osteogenesis coupling. During bone defect repair, type H vessels identified by high expression of CD31 and EMCN played a critical role in coupling bone formation and regeneration.^39,40^ Bone defect in cKO mice had fewer type H vessels, but mechanical loading partially rescued vessel volume to normal levels (Figs. 7f, h, S10a, b). Moreover, the low number of osteoprogenitors (Sca-1^+^) and osteoblasts (OSX^+^) induced by periostin deficiency returned to normal levels as in WT mice without loading (Fig. 7g, i, j). We also investigated the effect of mechanical loading on angiogenesis-osteogenesis coupling in periostin cKO mice. The distance between each OSX^+^ cell and the nearest type H vessel was calculated (Figs. 7k, S10c). WT-loaded tibia showed the highest number of OSX^+^ cells in contact with type H vessels, followed by WT-control tibia and cKO-loaded tibia. The lowest number of OSX^+^ cells in contact with vessels was in the cKO control tibia (Figs. 8l, S10d, e). These results demonstrated that in the absence of myeloid cell-secreted periostin, mechanical loading alleviated the negative effects on bone repair by promoting angiogenesis-osteogenesis coupling.

### Implantation of mechanically conditioned macrophages improved bone defect repair

To elucidate the mechanisms by which mechanical stimulation enhanced periostin expression in macrophages, we performed RNA-sequencing (RNA-seq) analysis of the control and loaded bone defects. RNA-seq analysis of mouse tibia showed that 317 genes were upregulated and 308 genes were downregulated in mice tibiae in response to mechanical loading (Fig. S11a). Pathway analysis of differentially expressed genes (DEGs) identified enrichment in extracellular matrix organization, extracellular matrix structure, and collagen-containing extracellular matrix after mechanical loading (Fig. S11b). The KEGG signaling pathway enrichment analysis of DEGs in the RNA-seq data showed that the activated pathways in loaded group were associated with immunity, including TGF-β, T cell receptor, and Relaxin signaling pathway (Fig. S11c). Fluid shear stress (FSS) increased the mRNA and protein expression of periostin in BMDMs, as measured by RT-PCR and ELISA (Fig. 8a, b). This finding was further corroborated by the fluorescence intensity of periostin (Fig. S12a). In addition, FSS increased the expression of total and active TGF-β in BMDMs as measured by ELISA (Fig. S12b). We speculated that TGF-β induces the secretion of periostin. Indeed, TGF-β upregulated periostin expression in BMDMs at non-cytotoxic concentrations of 10 ng/mL (Figs. S12c, 8c). These results indicated that mechanical stimulation activated TGF-β and upregulated periostin expression in macrophages. To investigate the activation of TGF-β under FSS stimulation, we observed nuclear localized p-Smad2/3 during FSS stimulation and found most of BMDMs had activated Smad2/3 after 40 min and remained active in around 50% of cells after 12 h, suggesting FSS activated the TGF-β pathway (Fig. S12d).

To assess the role of mechanically stimulated periostin expression in macrophages in promoting bone defect repair, we transplanted mechanically conditioned macrophages into the MTD with the surrounding periosteum removed. We first isolated BMDMs from C57BL/6J mice and seeded them onto an artificial periosteum composed of polycaprolactone/hydroxyapatite (PCL/HA), which has been used in bone tissue engineering for its ability of cell proliferation and bone growth.^41,42^ PCL/HA is mechanically flexible and has low cytotoxicity and high biocompatibility in cell adhesion and proliferation (Fig. S13a–e). The cellularized artificial periosteum was treated with FSS or TGF-β and transplanted into the defect site (Fig. 8d). FSS promoted M2 macrophage polarization and increased periostin expression (Fig. S13f). TGF-β-treated BMDMs were used as a positive control. Transplantation of FSS- or TGF-β-treated BMDMs induced more new bone accrual compared with nontreated BMDMs, with higher BV/TV (Fig. 8e). FSS- and TGF-β-treated BMDMs produced similar effects, indicating an equivalent effect of these two approaches in bone regeneration (Fig. 8f). To further verify the contribution of FSS-stimulated macrophages to bone regeneration, we measured the expression of periostin, blood vessels and osteoblasts in the defect site on PSD 10. Transplantation of FSS- or TGF-β-treated BMDMs induced higher expression of OSX^+^ cells, EMCN^+^ vessels, and periostin compared to non-conditioned BMDMs (Fig. 8f). The angiogenesis-osteogenesis coupling in the defect site was also enhanced after transplantation of FSS-treated BMDMs (Fig. 8f). Similar changes of higher expression of periostin, OSX^+^ cells, and EMCN^+^ vessels were present in subcutaneous transplantation of FSS-treated BMDMs compared with non-conditioned BMDMs (Fig. S14c, d). These results suggested that mechanically-conditioned macrophages promoted bone regeneration, and may be a potential therapeutic approach for bone defect repair.

## Discussion

Fracture healing was a complex and dynamic process requiring coordinated interaction of various systems to restore the structural integrity and the load-bearing capacity of the newly formed bone. The immune system acted as a crucial regulatory component that played an essential role in all stages of bone regeneration.^43^ However, the specific sources and mechanisms of immune cells that participated in bone repair processes remained poorly elucidated. Macrophages, a ubiquitous immune cell population, have been widely studied in various disease models including tumors, cardiovascular diseases, and respiratory disease.^44^ In our study, we have identified that macrophages, specifically those located in the periosteum, were actively involved in the complete process of healing, with a persistent expression of periostin. Immunofluorescence and FACS analysis revealed an augmented level of periostin in M2 macrophages as opposed to M1 macrophages. The depletion of periostin in macrophages has resulted in impaired bone modeling and remodeling due to the failure of periostin to facilitate the polarization of M2 macrophages and the ossification of osteoblasts. Additionally, previous studies have emphasized the crucial influence of the mechanical environment on osteogenesis and bone remodeling by regulating the differentiation and function of osteoblasts.^45,46^ Our findings demonstrated that, apart from osteoblasts, macrophages within the bone possessed the ability to react to mechanical stimuli. Mechanical stimulation activated the periostin expression in macrophages, leading to the modulation of M2 macrophage polarization. The disruption of periostin function abolished the regulation of macrophage polarization by mechanical stimuli. However, this deficiency was compensated for the impact on bone regeneration by promoting angiogenesis-osteogenesis coupling. Consequently, mechanically stimulated macrophages might co-regulate bone defect repair through increasing periostin expression and polarization towards the M2 subtype. Notably, the transplantation of mechanically conditioned macrophages into the injury site significantly increased bone repair, offering a promising approach for bone repair.

The immune response was a well-organized process with distinct cellular activities throughout all phases of bone regeneration. LysM was expressed in myelomonocytic cells, including monocytes, macrophages, and granulocytes in mice Initially, neutrophils were recruited to the fracture site to eliminate pathogens and cellular debris.^47,48^ Subsequently, M1 macrophages were recruited by neutrophils to form a hematoma, and the hypoxic and acidic environment created by the hematoma triggers the expression of calcium phosphate and ALP, thereby promoting M2 macrophage polarization and osteogenic differentiation.^49,50^ Macrophages involved in bone repair have traditionally been divided into M1 and M2 types induced by Th1 and Th2 cytokines. As research continues, M2 macrophages have shown more subtypes, including M2a, M2b, M2c, and M2d.^37^ M2a macrophages secreted profibrotic factors that aid tissue repair, while M2b released pro- and anti-inflammatory cytokines with immunomodulatory effects. M2c macrophages were associated with immunosuppression and regulated extracellular matrix remodeling, whereas M2d were associated with tumors and vascularization.^51–53^ All M2 macrophages possessed a range of anti-inflammatory factors, and there was functional overlap between subtypes. Due to the high phenotypic plasticity of macrophages, the M1/M2 dichotomy cannot fully capture their functional diversity, and subtypes related to bone repair can be subsequently explored through more defined markers. Ongoing studies have identified CD169^+^ osteomas, CD166^+^ osteomas, and osteoclasts as modulators of bone homeostasis.^54^ Inflammatory factors facilitated bone repair in the initial stages of fracture healing, but their prolonged presence could disrupt the balance of the M1-to-M2 transition, impeding the process of cartilage formation, angiogenesis, and osteogenic differentiation.^55,56^ Depletion of macrophages led to early skeletal growth retardation and osteoporosis accompanied by a decrease in bone mineral density and trabecular numbers.^3^ Bone-resident macrophages, or osteomacs, were near osteoblasts at the bone surface and significantly influenced the osteogenic differentiation of MSCs.^57^ These interactions occurred through direct cell-to-cell contacts or indirect immunomodulator secretion. For instance, when co-cultured with MSCs, M2 macrophages enhanced ALP activity and bone mineralization, whereas M1 macrophages induced the opposite effect.^13^ IL-10 and IL-4 secreted by macrophages played a pivotal role in recruiting MSCs to the site of injury, stimulating MSC osteogenic differentiation, and promoting new bone formation.^58,59^ In turn, MSCs secreted IL-10 to mediate the M2 macrophage polarization under pro-inflammatory conditions.^60^ However, in response to inflammatory cytokines TNF-α and IL-1α, MSCs released anti-angiogenic factors that impeded endothelial cell proliferation and migration, thus inhibiting bone formation.^61^ Thus, the interaction of macrophages and osteoblasts jointly regulated bone regeneration. In addition to these inflammatory factors, we found that periostin could also mediate the communication between macrophages and osteoblasts. Macrophage-derived periostin induced the expression of osteoprogenitors and promoted osteoblast differentiation and mineralization. At the same time, in vivo implantation of macrophages enhanced new bone formation at the site of injury, further illustrating the regulatory role of macrophages in osteogenesis.

Mechanical forces regulated macrophage activities such as proliferation, migration, polarization, and phagocytosis. A distraction osteogenesis model in mice showed a significantly increased number of macrophages.^62^ In vivo, FSS could lead to macrophage infiltration and modulation of tumor inflammation.^63^ In vitro, extracellular stiffness enhanced cytokine secretion and phagocytic activity in macrophages.^64^ Our findings revealed that during bone regeneration, mechanical loading promoted the accumulation of macrophages at bone defects, particularly M2 macrophages involved in repair and remodeling, with enhanced expression of periostin. However, the M1 polarization induced by mechanical stretching may be related to the direction and magnitude of the force.^65^ Current research on the mechanical regulation of macrophages primarily focused on their cell function. In molecular mechanisms, mechanosensitive YAP has been implicated in exacerbating inflammation through the regulation of M1/M2 macrophage polarization.^66^ Additionally, periostin has been shown to regulate M2 macrophage polarization in myocardial infarction and cancer.^67^ We demonstrated that periostin regulated macrophage polarization in the skeletal system. The depletion of periostin in macrophages did not affect their ability to polarize towards the M1 subtype but impaired the polarization of the M2 subtype. The mechanical sensitivity of macrophages was compromised in the absence of periostin, diminishing the impact of the mechanical environment on M2 macrophage polarization. Furthermore, we also observed that FSS enhanced periostin expression in macrophages with concomitant increase in nuclear p-Smad2/3. The transplantation of mechanically conditioned macrophages into the injured site resulted in an increased formation of new bone, thereby accelerating the process of bone healing. Therefore, periostin could serve as one of the signaling molecules involved in the mechanical regulation of macrophages.

The periosteum was a complex and dynamic connective tissue membrane that enveloped the bone surface, housing a diverse population of specialized cells crucial for bone growth and repair. These cells included bone marrow mesenchymal stem cells (BMSCs), skeletal stem cells (SSCs), fibroblasts, pericytes, and macrophages, all working in synergy to orchestrate the intricate processes of bone regeneration.^68^ The indispensability of the periosteum in bone healing became evident when it was stripped or removed, as impaired bone repair and delayed union of fractures were observed.^69^ Due to its location on the outer surface of bones, the periosteum exhibited remarkable sensitivity to mechanical stimuli. When subjected to mechanical tension or increased loading, the periosteum exhibited a dynamic response, characterized by observable chondral growth and new bone generation.^70^ The expression of periostin was predominantly in a distinct population of periosteal cells (PCs). The disruption of periostin function hampered the reconstitution of PCs following a fracture, highlighting the presence of critical PCs within the periosteum and the essential role of periostin in sustaining bone structure and function.^17^ These findings underscored not only the critical nature of the PCs within the periosteum but also emphasized the essential role of periostin in the maintenance of bone structure and function. Our investigations revealed a notable presence of periostin expression in macrophages, particularly those localized within the periosteum as a constituent PC pool. These macrophages exhibited a substantial capacity to contribute to bone repair. Notably, when the original periosteum was removed, a consequential delay in bone repair was observed. However, upon re-implantation of macrophages into the periosteal region, the adverse effects stemming from periosteal removal were restored, highlighting the critical role of macrophages in mitigating the negative consequences associated with periosteal disruption. Thus, macrophages emerged as significant contributors within the PC pool, serving as an important source for periostin production and actively participating in the reparative mechanisms for bone defects.

In summary, we have uncovered a significant role of macrophage-derived periostin in the repair of bone defects. Periostin functioned as an effector protein in macrophages in response to mechanical stimuli. Particularly, M2 macrophages residing in the periosteum were identified as a major source of periostin. Mechanical stimulation enhanced periostin expression in macrophages and exerted a dual effect by inducing M2 macrophage polarization and concurrently facilitating osteoblast differentiation. Consequently, this signaling network collaboratively expedited the process of bone healing in response to mechanical stimulation. Together, these findings demonstrated periostin expression in macrophages was a mechanosensitive phenomenon, and had a significant impact on bone regeneration, highlighting the potential utility of mechanically conditioned macrophages in orthopedic applications.

## Materials and methods

### Mice

All procedures were performed by accordance with the “Guide for the Care and Use of Laboratory Animals” and in compliance with the relevant ethics policy and the requirements of the Ethics Committee of Southern University of Science and Technology (certificate SUSTCJY20190427). All mice were maintained in a C57BL/6J background. Specific pathogen-free (SPF) twenty-week­old female C57BL/6J mice were purchased from Byneng Biotechnology Co., Ltd. (Guangzhou, China). LysM-Cre mice were generously provided by Professor Ling Guo (Cancer Hospital Chinese Academy of Medical Sciences Shenzhen Hospital). ROSA 26 mice (No. 007914) and *POSTN* ^*fl/fl*^ mice (CKOCMP-50706-Postn-B6J-VA, Cyagen Biosciences, Inc.) were purchased from Jackson Laboratory. ROSA mice were crossed with *LysM-Cre* mice to obtain *LysM-Cre-tdTomato* (tdTomato) mice. To generate *LysM-Cre; POSTN*^*−/−*^ (cKO) mice, *POSTN* ^*fl/fl*^ mice were mated with LysM-Cre mice to obtain *LysM-Cre; POSTN* ^*fl/+*^ mice, which were then mated with *POSTN* ^*fl/fl*^ mice. To confirm knockout efficiency, littermate analysis was performed by investigators blinded to the mouse genotype. All mice were bred under SPF conditions and weighed every two weeks.

### Tibial defect model

Mice bone defect repair was modeled by creating a monocortical defect on the anteromedial surface of the tibia. In brief, mice were anesthetized via inhalation of 2.5% isoflurane and subcutaneously injected with meloxicam. The skin and muscles on the surface of the mouse tibia were peeled off to expose the tibia, and a hole with a diameter of 1 mm was drilled on the anteromedial surface of the tibia using a high-speed drill. The drilling site was flushed with saline to remove bone debris. Then the surrounding muscles and skin were sutured, and erythromycin ointment was applied. Mice were placed on a heating plate until they were awake and able to move normally.

### Mechanical loading

On postsurgical days (PSDs) 5, 6, 7, and 8, mechanical loading was applied to the left tibia of the mouse using an electromechanical testing system (ElectroForce 3200 system, TA Instruments, USA). The force applied to wild-type (WT) and tdTomato mice was 6 N peak, 2 Hz sinusoidal, 120 cycles per day. Forces of 2 N, 3 N, 4 N, 5 N, 6 N, and 8 N peak, 2 Hz sinusoidal, 120 cycles/day forces were applied to the tibia of cKO mice, resulting in the same micro-strain in cKO mice as in WT mice. The right tibia served as an unloaded control. All mice were sacrificed on PSD 10.

### Immunofluorescence staining

Fresh tibiae were fixed in 4% paraformaldehyde at 4°C for 4 h and decalcified in 0.5 mol/L EDTA at 4 °C for 24 h. The tibiae were cryoprotected in 20% sucrose solution at 4°C for 24 h, then embedded in a gelatin-based medium and stored at −80 °C. Tibiae were cryosectioned into 80 μm thick tissue slices along the long axis of the tibia, perpendicular to the plane of the defect for the creation of longitudinal sections with a cryostat (Leica CM1950, Weztlar, Germany). The samples were stained with primary antibodies against OSX (1:200; ab22552, Abcam, Cambridge, UK), F4/80 (1:200; ab6640, Abcam, Cambridge, UK), periostin (1:200; AF2955, R&D Systems, Minneapolis, MN, USA), CD31 Alex Fluor 488 (1:200; FAB3628G, R&D Systems, Minneapolis, MN, USA), iNOS (1:200; ab3523, Abcam, Cambridge, UK), CD206 (1:200; PA5-46994, Thermo Fisher Scientific, Waltham, MA, USA), Sca-1 (1:200; 710952, Thermo Fisher Scientific, Waltham, MA, USA), Ki-67 (1:200; 710952, Abcam, Cambridge, UK) and endomucin (1:200; sc­65495, Santa Cruz Biotechnology, USA) followed by Alexa Fluor secondary antibodies from donkey (1:400; Thermo Fisher Scientific, Waltham, MA, USA). The slides were mounted with DAPI Fluoromout­G (0100-20, SouthernBiotech, Birmingham, AL, USA) and sealed with coverslips.

### Confocal and two-photon microscopy imaging

Three-dimensional fluorescent images were acquired with a 20× objective lens of ZEISS LSM 980 confocal microscope (Germany). The Z­stacks (40 μm thick) were taken at a size of 1 024 × 1 024 pixels, x­y resolution of 0.624 mm, and z­step of 2 mm. The 1 mm defect was imaged by tiling three Z­stacks, spanning 1 500 mm along the long axis of the tibia from one side of the intact cortical bone to the other. DAPI images were acquired (425–475 nm filter) with 405 nm excitation, F4/80, CD31, sca-1 and Ki67 images were acquired (500–530 nm filter) with 488 nm excitation, Emcn, CD206, periostin and tdTomato images were acquired (552–617 nm filter) with 594 nm excitation, OSX, iNOS, periostin images were acquired (662–737 nm filter) with 647 nm excitation, and the second harmonic generation (SHG) of collagen fibers images were acquired (420–465 nm filter) with 860 nm excitation. The imaging was analyzed by Imaris software (Bitplane, USA).

### Spatial correlation analysis

Distances between OSX^+^ cells and type H vessels were calculated as described previously.^71^ For each channel, a 3D-rendered image and its corresponding distance mask were created in the Imaris software. The distance mask encoded the distance of each pixel to the nearest surface of the channel as the brightness value of the pixel. The diameters of osteoprogenitors were approximately 2 0­50 μm. For the accurate estimation of the spatial correlation between cells and periostin, 10 μm was selected as the boundary condition. When the distance between OSX^+^ cell and type H vessel was less than 10 μm, OSX^+^ cells were determined to be coupled with vessels. The distance between OSX^+^ cells and vessels was then calculated by mapping the encoded distance values from one channel to the spatial coordinates of the other channel. Similarly, the distance between F4/80^+^ cells and periostin was also calculated as above. In brief, the 3D-rendered images and distance masks were created for F4/80 and periostin, respectively. The distance between F4/80^+^ cells to periostin was calculated by mapping the encoded distance values from one channel to the spatial coordinates of the other channel. When the distance between F4/80^+^ cells and periostin was less than 10 μm, periostin was determined to be secreted by this cell.

### Microcomputed tomography

Freshly tibiae and femurs were dissected and stored in PBS at 4°C. These specimens were scanned at 6 μm resolution with a microcomputed tomography (Micro-CT) system (Skyscan 1172, Bruker, USA). The intact bone and defect regions were reconstructed using CTvox software (Materialize, USA). Newly formed bone within the defect was quantified for various parameters, including percent bone volume (BV/TV), trabecular number (Tb. N), trabecular thickness (Tb.Th), crossectional bone area per tissue area (B.Ar/T.Ar), maximum moment of inertia (MMI [max]), and connectivity density (Conn. Dn).

### Strain field measurement

The mechanical properties of mice tibiae were measured using DIC according to established methods.^72^ The revealed periosteal region of the tibia was coated by a layer of matte, water-soluble white acrylic paint (XF-2, Tamiya Paint, USA). Minute black speckles were then applied using a high-precision airbrush with matte black water-based paint (0741, Haoshun, China). The painted tibiae were then placed on the loading receptacles of an electromagnetic mechanical test system. The test was conducted at a rate of 0.5 N/s up to a maximum axial load of 12 N. The tibiae were captured in the DIC system (XTDIC-Micro, XTOP, China). The strain distribution was calculated by XTDIC software.

### Finite element analysis

The mouse tibia geometry data was imported into MIMICS 21.0 software to reconstruct a 3D solid (Materialize, Leuven, Belgium). The optimized 3D model of cortical bone was imported to Solidworks software 2018 (Dassault Systemes S.A, USA). A 1-mm circular defect was created on the anterior surface of the mouse tibia to simulate the regenerating tissue within the defect. The 3D model of the tibia assembly was imported into Abaqus software 6.1 (Dassault Systèmes Simulia Corp, Providence, RI). In this model, the cortical bone was assigned material properties of Young’s Modulus of 17 GPa and Poisson’s ratio of 0.3. The regenerating tissue was assigned Young’s Modulus of 0.3 GPa and Poisson’s ratio of 0.3. Tied contact was used to connect regenerating tissue within the circular hole on the cortical bone. Boundary conditions and loads were chosen to match the in vivo experimental conditions. The distal end of the tibia was completely fixed. Axial loading of 4 N or 12 N was applied on the metaphyseal surface of the tibia. After finishing the finite element models, the main outcomes of von Mises stress and the strains along the loading axis on the tibial cortical bone surface were calculated using Abaqus.

### Four-point bending test

Femurs from WT and cKO mice were harvested and stored in PBS at −20 °C. Samples were returned to room temperature and fixed on an electromagnetic dynamic mechanical testing system (M-1000, CARE, China). This system utilized a 100-N load cell and a linear variable displacement transducer with a ± 40 mm maximum range. The four-point bending mold had a 4 mm upper- and 8 mm lower-span width. The parameters of bending modulus (GPa), ultimate strength (MPa), maximum load (N), and yield load (N) were calculated via a mapped displacement-loading curve.

### Dynamic histomorphometry

Mice were intraperitoneally injected with calcein (5 mg/kg) on PSD 2 and 9 after MTD surgery. Tibiae were collected and embedded into methyl methacrylate as previous study.^36^ The images were captured using confocal microscopy with 488 nm excitation. Dynamic histomorphometry data were measured for periosteal surfaces. The calculation formulas included mineral apposition rate (MAR) and bone formation rate (BFR).

### Flow Cytometry Analysis

Fresh tibiae of C57BL/6 J mice were collected and cut 1 mm above and below the defect on PSD 10. Bone marrow cells were flushed from the cut ends of tibiae with complete media (DMEM containing 10% FBS and 1% penicillin­ streptomycin). The cell suspension was then filtered through a 70-mm filter mesh. After centrifugation the cell supernatant, the red blood cells were lysed according to the instructions (C3702, Beyotime, CN). Cells were resuspended in 100 μL FACS buffer (PBS containing 1 nmol/L EDTA and 0.1% BSA). For analyzing M1 and M2 macrophages, cells were incubated with BV421 Rat anti-mouse F4/80 antibody (1:200, 565411, BD Biosciences, San Jose, CA) and PE-Cy7 Rat anti-mouse CD86 (M1 macrophage marker, 560582, BD Biosciences, San Jose, CA) for 30 min at 4 °C, followed by fixation and permeabilization. Alexa Fluor 647 Rat anti-mouse CD206 (M2 macrophage marker, 565250, BD Biosciences, San Jose, CA) was then used for intracellular staining. For analyzing periostin^+^ macrophage, cells were incubated with anti-periostin antibody (1:200; ab14041, Abcam, Cambridge, UK), followed by Alexa Fluor 488 secondary antibodies from donkey (1:400; Thermo Fisher Scientific, Waltham, MA, USA). Cells were then washed with FACS buffer and analyzed using a four-laser cell sorter (BD FACSCanto SORP, BD Biosciences, San Jose, CA). Flow cytometry data was analyzed with FlowJo software (version 10, BD Bioscience).

### RNA-seq and analysis

Total RNA was extracted from mouse defect site using Trizol reagent following the manufacturer’s instructions. MGISEQ-2000RS Kit for DNBseq 2000 was used to purify poly-A^+^ transcripts and generate libraries with multiplexed barcode adapters following the manufacturer’s instructions. High-throughput sequencing (150 bp, two paired-end) was performed using the DNBseq 2000 in the BGI Genomics Co.,Ltd. All samples passed quality control analysis using Fast QC (Agilent). RNA-seq reads were aligned to the mouse genome (GRCm39) using HISAT2 with default parameters. DESeq2 utilized reads for counts per million (CPM), adjusted *P*-values (adj. *P*), and log_2_ fold changes (Log_2_FC) computation. Genes with *P* value < 0.05 and fold-change of at least 1.5 were identified as differentially expressed genes (DEGs) between conditions using the DESeq2 analysis of three RNA-seq biological replicates from different donors. Integrated pathway analysis was performed using Kyoto Encyclopedia of Genes and Genomes (KEGG) and gene ontology (GO) pathways in clusterProfiler. *P* and False discovery rate (FDR) values were calculated following the R program’s instructions.

### Cell cultures

Mouse fibroblasts (L929) were purchased from Procell Life Science & Technology Co., Ltd. L929 cells were cultured in DMEM medium containing 10% FBS and 1% penicillin/streptomycin. The L929 cell culture supernatant was collected and filtered through a 0.22 μm membrane filter for culturing BMDMs. BMDMs were extracted from mice tibiae and femurs. In brief, mice were sacrificed and soaked in 75% ethanol. The skin and muscle surrounding bones were removed and kept on ice in PBS containing 1% penicillin/streptomycin until further dissection. A 1-mL syringe filled with growth medium (DMEM+ 20% FBS+ 1% penicillin/streptomycin+ 20% L929 cell culture supernatant) was inserted into the marrow cavity. The cells were then cultured in a growth medium for 7 days to harvest BMDMs.

### Macrophage polarization

BMDMs were plated in 12­-well plates at the density of 4 × 10^4^ cells/well in growth media. After overnight incubation, the growth medium was replaced with fresh growth media and maintained until the cell reached over 80% confluency. The medium was changed every 2 days. LPS was used to induce macrophage polarization to the M1 subtype, and IL-4 protein was used to induce macrophage polarization to the M2 subtype. As previous instruction, the culture medium was changed into M1 macrophage polarization media containing 100 ng/mL LPS (L2880, Sigma-Aldrich, German), the matured M1 macrophages were then harvested at 24 h. For M2 polarization, the culture medium was replaced with M2 macrophage polarization media containing 20 ng/mL IL-4 (PeproTech, Rocky Hill, NJ). Matured M2 macrophages were harvested at 24 h.

### Fluid shear stress

BMDMs were plated in a 6­-well plate at a density of 3 × 10^5^ per well in a growth medium. After overnight incubation, cells were exposed to orbital shear stress on the shaker at 1 Pa for 2 h. BMDMs were seeded on round coverslips for immunofluorescence staining. BMDMs in statics status were used as the control.

### Real-time PCR

Mice tibiae were collected immediately after euthanasia, frozen in liquid nitrogen, and ground into powder. Total RNA from BMDMs and mouse tibiae were extracted by Trizol reagent (15596, Thermo Fisher Scientific, Waltham, MA, USA). The RNA was transcribed into cDNA using the RevertAid First Strand cDNA synthesis kit (K1622, Thermo Fisher Scientific, Waltham, MA, USA). Quantitative RT-PCR was performed using ABI QuantStudio 1 (A40426, Thermo Fisher Scientific, Waltham, MA, USA) using GoTaq® qPCR Master Mix (A6002, Promega, Madison, WI, USA). The primer sequences were displayed in Supporting Information (Table 1). The results were calculated as the 2^−ΔΔCt^ method and normalized to the expression levels of the housekeeping gene 18S.

### Western blotting

Total protein was extracted following the instructions for RIPA lysis buffer (P0013B, Beyotime, CN) in BMDMs. The concentration of total protein was measured using the BCA Protein Assay Kit (P0012, Beyotime, CN). Proteins were separated by sodium dodecyl sulfate-polyacrylamide gel electrophoresis (SDS-PAGE) and then transferred to the nitrocellulose blotting membranes (MilliporeSigma, Burlington, MA). The primary antibody for Periostin (1:1000, ab14041, Abcam, Cambridge, UK), Smad2 (1:1 000, 5339S, Cell Signaling Technology, Danvers, MA), Smad3 (1:1 000, 9523S, Cell Signaling Technology, Danvers, MA), p-Smad2 (1:1 000, 18338S, Cell Signaling Technology, Danvers, MA), p-Smad3 (1:1 000, 9520S, Cell Signaling Technology, Danvers, MA), and β-Actin (1:1 000, 4970S, Cell Signaling Technology, Danvers, MA) was applied for incubation, followed by anti-Rabbit IgG (H+L) Secondary Antibody, HRP conjugate (1:10 000, 31460, Thermo Fisher Scientific, Waltham, MA, USA). The imaging was detected using Fusion SoloS.EDGE Chemiluminescence Imaging (Vilber Bio Imaging, France).

### Scanning electron microscopy

BMDM morphology on the PCL/HA membrane (provided by Yan Wang, Southern University of Science and Technology) was analyzed using scanning electron microscopy (SEM). BMDMs were seeded on the PCL/HA membrane in a 6­well plate at a density of 3 × 10^5^ per well. After 48 h incubation, samples with BMDMs were washed with double distilled water, fixed with 4% paraformaldehyde at room temperature, dehydrated in a graded ethanol solution, and sputter-coated with gold. The morphology of BMDMs was then observed by a scanning electron microscope (Regulus 8100, Hitachi, Japan).

### Cell viability

The biocompatibility of the PCL/HA membrane was tested by seeding BMDMs on the membrane in a 96-well plate at a density of 1 × 10^4^ cells per well. After 48 h incubation, tetrazolium salt-based colorimetric assay (CCK8 test) was performed according to the instruction (CCK8 kit, Dojindo, Japan). The absorbance of each well was measured by a microplate spectrophotometer (Bio-Tek, Winooski, VT, USA) at 450 nm.

### TGF-β concentration evaluation

BMDMs were seeded in a 96-well plate at a density of 1 × 10^4^ cells per well. After incubation overnight, cells were cultured in a growth medium containing 0, 2.5, 5.0, 10.0, 20.0, 40.0, 80.0, or 160.0 ng/mL of TGF-β for 24 h. The non-cytotoxic concentration was determined by CCK8 assay.

### Artificial periosteum transplantation

PCL/HA membranes served as artificial periosteum. BMDMs extracted from C57BL/6J mice were seeded on PCL/HA membranes. C57BL/6J mice with MTD surgery as recipient mice were randomly divided into four groups: (1) defects for PCL/HA as blank controls; (2) defects for implantation of PCL/HA+BMDM; (3) defects for implantation of PCL/HA+BMDM-FSS; (4) defects for implantation of PCL/HA+BMDM-TGF-β. After MTD surgery, the periosteum around the mice defect was excised, and the PCL/HA membranes in different groups were curved to cover the defect site. Two sides of the membrane were sutured together with the surrounding muscles. Mice were sacrificed on PSD 10, and tibiae were collected for further investigation.

### Subcutaneous implantation

PCL/HA membranes seeded with BMDMs, BMDMs-FSS, and BMDMs-TGF-β implanted subcutaneously, with PCL/HA membranes used as control. Mice were anesthetized by inhalation of 2.5% isoflurane and subcutaneously injected with meloxicam. After disinfection with povidone-iodine, a 1 cm dorsal midline incision was made over the thoracolumbar area. Each membrane was inserted on one side of the incision. All the incisions were sutured, reinforced with wound clip, and received erythromycin ointment. The membranes in different groups were removed for the following study.

### Statistical analysis

Experimental data involving transplantations were validated using a minimum of six independent mice, and three separate cell groups. The presented data were aggregated from independent experiments. Statistical comparisons between the two groups were performed using a two-tailed Student’s t-test. Multiple comparisons with one variable were performed using two-tailed One-way ANOVA analysis with a Tukey’s Correction. Multiple comparisons with two or more variables were performed using two-tailed Two-way ANOVA analysis with a Tukey’s Correction. Data were presented as mean ± standard deviation (SD). Statistical significance was denoted as *P < 0.05, **P < 0.01, ***P < 0.001, and ****P < 0.000 1. Statistical analysis was performed using Prism 8 (GraphPad Software).

## Supplementary information

Supplementary data

Supplementary data - WB
