# Supplementary material for: Periostin+ myeloid cells improved long bone regeneration in a mechanosensitive manner
Source: Bone Res. 2024 Oct 15;12:59. doi: 10.1038/s41413-024-00361-5 (PMC11480347; doi:10.1038/s41413-024-00361-5)
Supplement: Supplementary file 1 — Supplementary data [file 41413_2024_361_MOESM1_ESM.docx]

**Periostin+ myeloid cells improved long bone regeneration in a mechanosensitive manner**

Ziyan Wang^1^, Minmin Lin^1^, Yonghao Pan^1^, Yang Liu^1^, Chengyu Yang^1^, Jianqun Wu^1^, Yan Wang^1^, Bingtong Yan^1^, Jingjing Zhou^1^, Rouxi Chen^3^,Chao Liu^1,2^*

^1^Department of Biomedical Engineering, Southern University of Science and Technology, Shenzhen, Guangdong, 518055, China

^2^Guangdong Provincial Key Laboratory of Advanced Biomaterials, Southern University of Science and Technology, Shenzhen, Guangdong, 518055, China

^3^ Department of Materials Science and Engineering, Southern University of Science and

Technology, Shenzhen, Guangdong, 518055, China

*Corresponding author

E-mail: liuc33@sustech.edu.cn

**This PDF file includes:**

Supplementary Figures 1 to 14

Supplementary Table 1


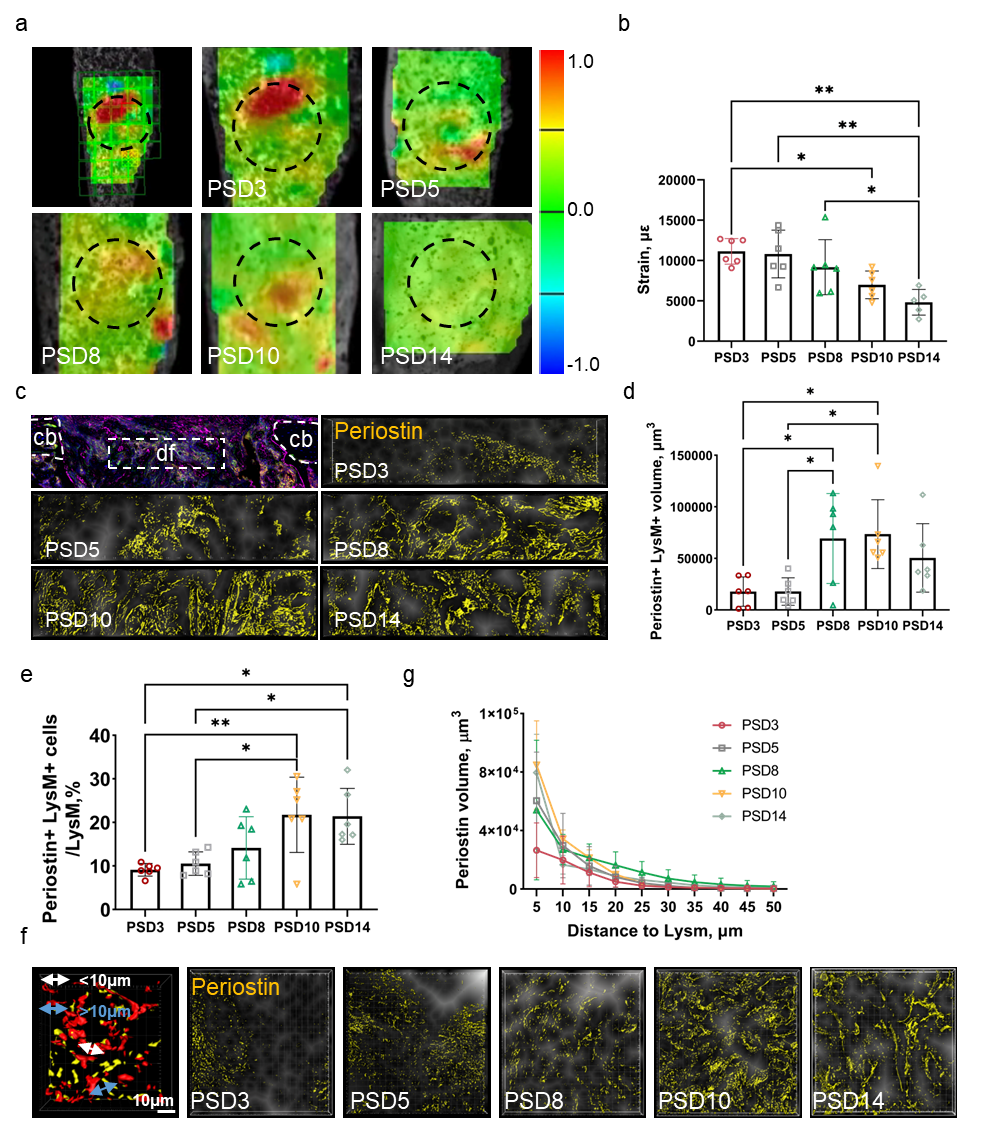


Supplementary Fig. 1 Periostin-expressing LysM+ cells mediated bone regeneration. **a** Distribution of microstrain within the defect site in tdTomato mice on PSD 3, 5, 8, 10, and 14. **b** Quantification of the strain in tdTomato mice. n = 6. **c** Spatial location of LysM+ cells and periostin in the defect site in tdTomato mice on PSD 3, 5, 8, 10, and 14. **d** Quantification of the periostin volume less than 10 microns from LysM+ cells in the defect site. n = 6. **e** Quantification of the ratio of periostin+ LysM+ cells in total LysM+ cells in throughout the defect site from colocalization analysis. n = 6. **f** Spatial location analysis of periostin and LysM+ cells throughout the defect site in tdTomato mice on PSD 3, 5, 8, 10, and 14. **g** Quantification of the periostin volume at different distance to LysM+ cells. n = 6. cb= cortical bone, df= bone defect, PSD= postsurgical day. **P* < 0.05; ***P* < 0.01; ****P* < 0.001; *****P* < 0.0001. Ordinary one-way ANOVA. Data were mean ± SD.


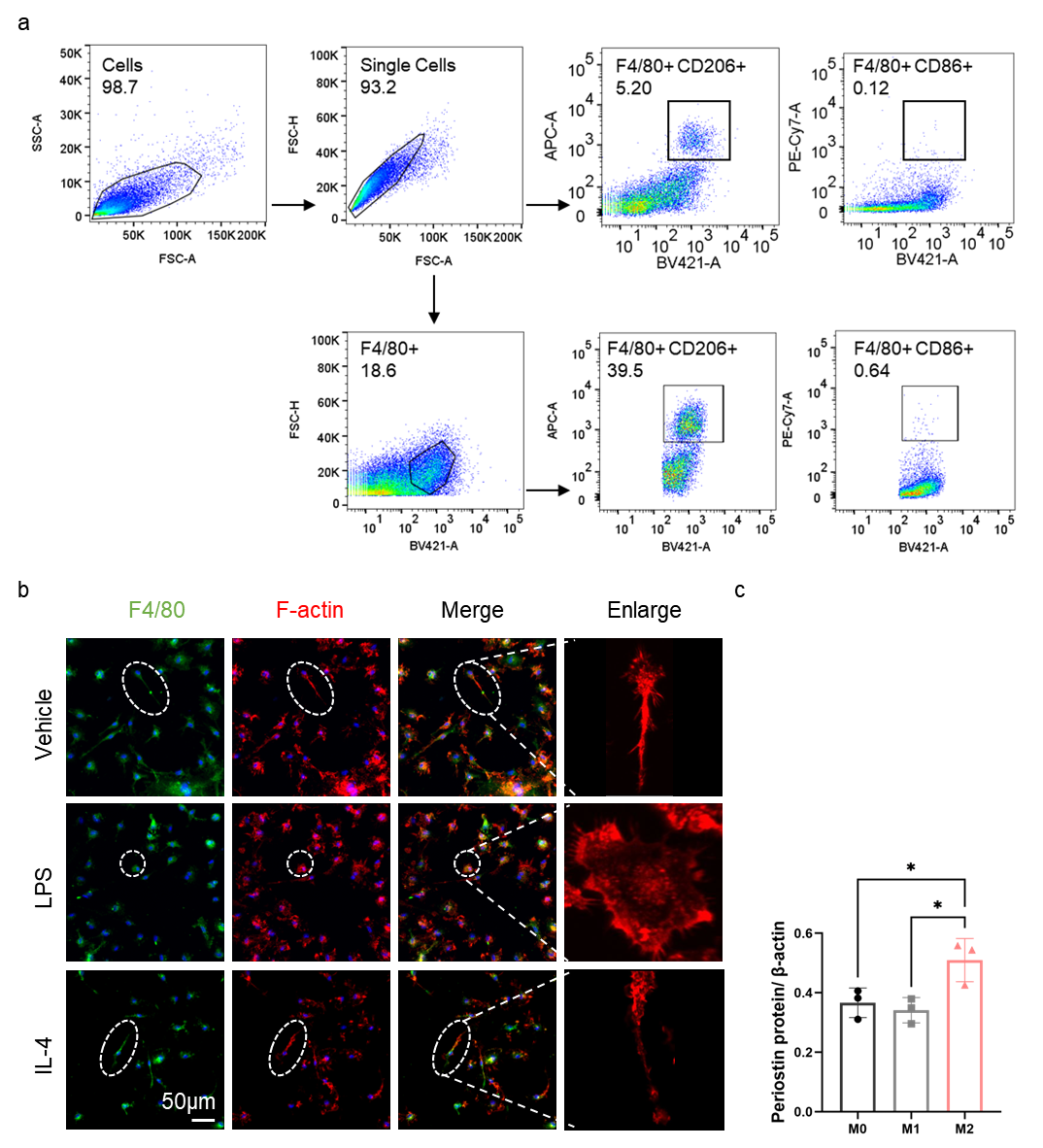


Supplementary Fig. 2 Induction of M1 and M2 polarization in macrophage. **a.** Flow cytometry analysis of the proportion of CD86+ F4/80+ cells and CD206+F4/80+ cells among all cells and macrophages. **b.** Immunofluorescence staining for the expression of F-actin and F4/80 within macrophages categorized into distinct subtypes: M0 subtype (vehicle-treatment), M1 subtype (LPS-treatment), and M2 subtype (IL-4-treatment). **c.** Quantification of western blot analysis of periostin expression in M0, M1, and M2. n = 3.


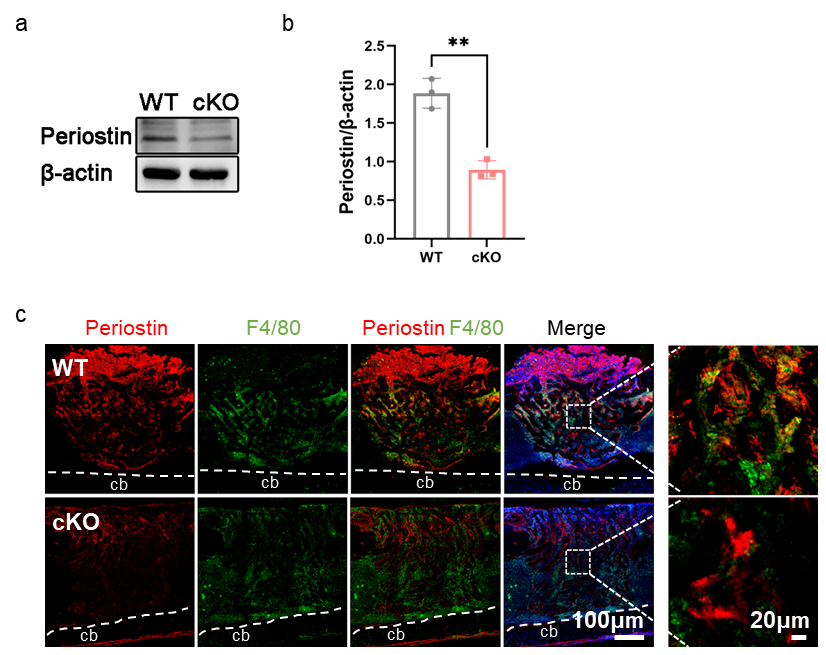


Supplementary Fig. 3 The validation of periostin knockout in macrophages. **a** Western blot analysis of the expression of periostin in BMDMs from WT or cKO mice. **b** Quantification of western blot analysis of periostin expression in BMDM cells from WT and cKO mice. n = 3. **c.** Immunofluorescence assay of the colocalization of periostin and F4/80 in WT or cKO mice.


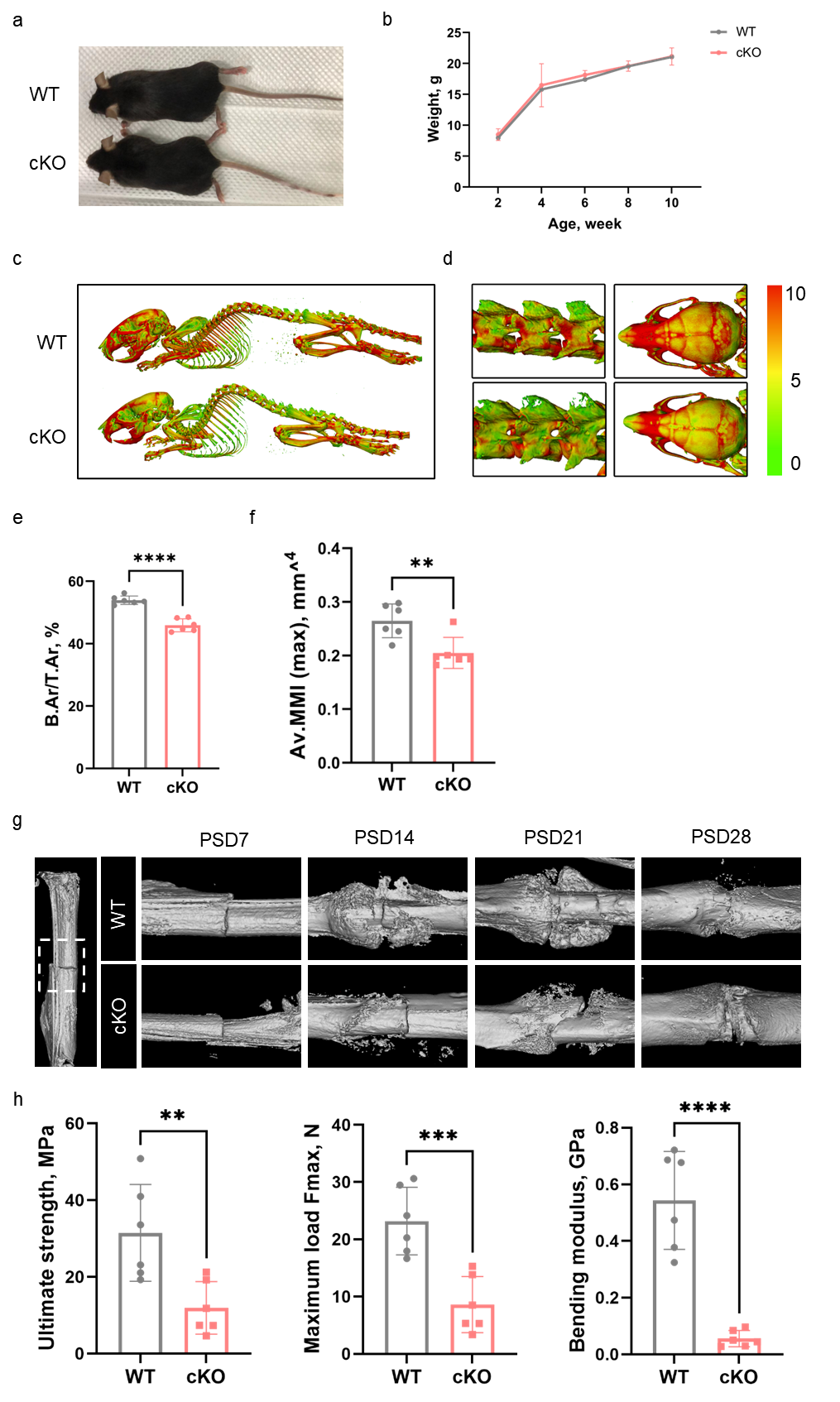


Supplementary Fig. 4 Knockout of periostin in myeloid cells led to diminished bone mass in mice. **a** Body sizes of WT or cKO mice at 12 weeks of age. **b** Body weight of WT or cKO mice from 2 to 12 weeks of age. **c-d** 3D Micro-CT images of (**c**) the whole-mount skeletal, and (**d**) spine and skull from WT or cKO mice. **e-f** Quantitative of the cortical bone Micro-CT parameters including, (**e**) B.Ar/T.Ar and (**f**) Av.MMI (Max) of WT or cKO mice. n = 6. **g.** 3D Micro-CT images of new bone accrual in the osteotomy model of WT or cKO mice. n = 6. **h.** Quantitative of four point-bending of mouse femur from WT mice and cKO mice under osteotomy surgery, including ultimate strength, bending modulus, and the maximum load. n = 6. ***P* < 0.01; *****P* < 0.0001. Student’s t test. Data were mean ± SD.


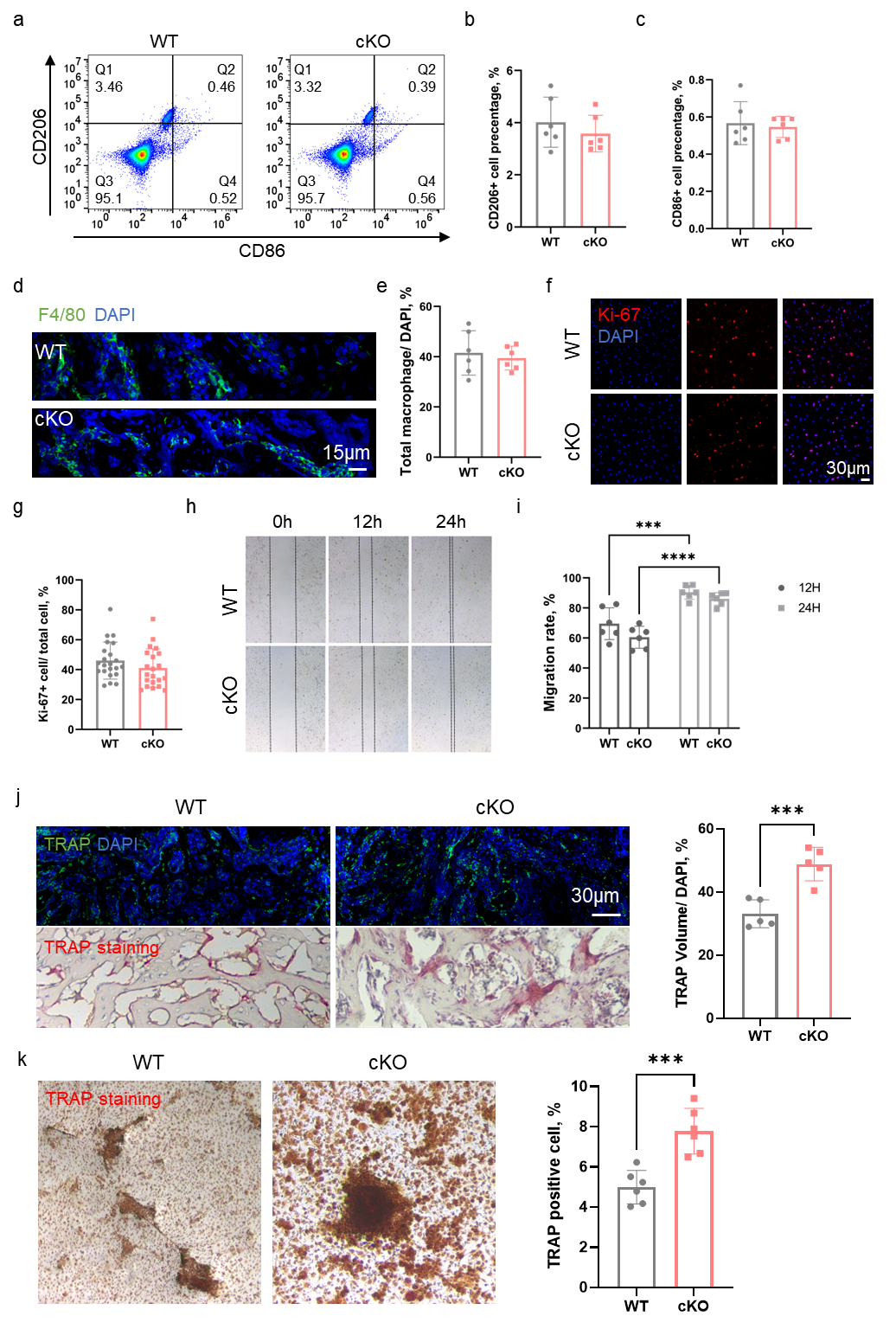


Supplementary Fig. 5 Knockout of periostin in myeloid cells did not impact macrophage proliferation and migration. **a** Flow cytometry analysis of CD86+ CD206- macrophages and CD86- CD206+ macrophages isolated from defect site in WT or cKO mice. **b-c** Quantification of the proportion of **(b)** CD206+ cells or **(c)** CD86+ macrophages in total cells. n = 6. **d** Immunofluorescence assay of the expression of F4/80 in the defect site of WT or cKO mice. **e** Quantification of the ratio of F4/80+ cells in WT or cKO mice. n = 6. **f** Immunofluorescence assay of Ki67+ BMDMs from WT or cKO mice. **g** Quantification of the ratio of Ki67+ BMDMs in WT or cKO mice. n = 6. **h** Cell scratch test results of BMDMs from WT or cKO mice after 0, 12, and 48 h in culture. **i** Quantification of the ratio of migration rate in BMDMs from WT or cKO mice. n = 6. **j.** Confocal imaging and histologic imaging of the healing bone defect by TRAP staining in WT mice and cKO mice and quantitative of the ration of TRAP+ cells total cells in WT mice and cKO mice. n = 5. **k.** TRAP staining and quantity of BMDM cells from WT and cKO mice n=6. ***P* < 0.01; ****P* < 0.001; *****P* < 0.0001. Ordinary two-way ANOVA or Student’s t test. Data were mean ± SD.


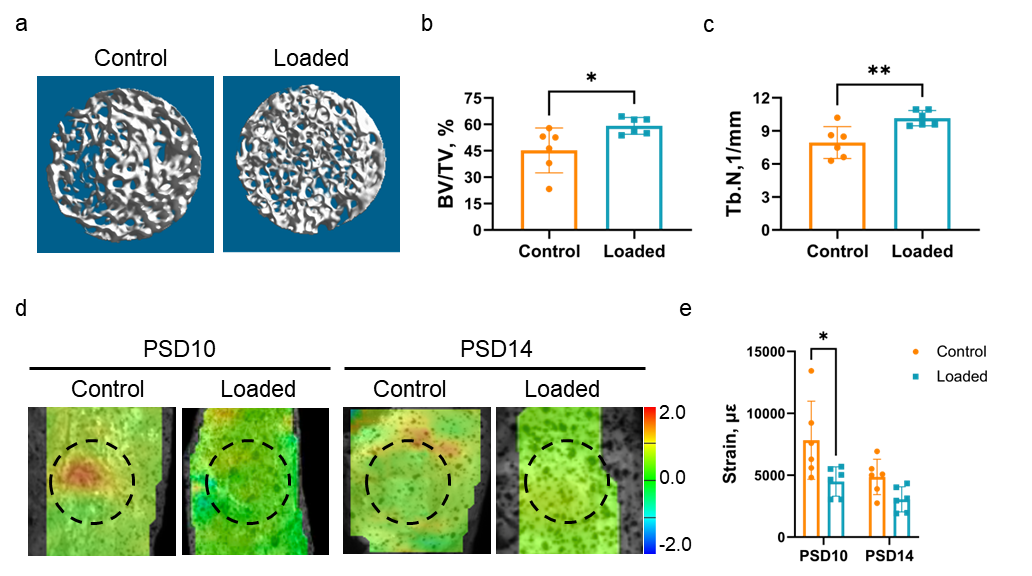


Supplementary Fig. 6 Mechanical loading increased bone formation and mechanical stiffness. **a** 3D Micro-CT images of new bone accrual isolated from WT mice on PSD 14 after MTD surgery in control and loaded tibia. **b-c** Quantitative parameters of Micro-CT analysis of new bone accrual including **(b)** BV/ TV and **(c)** Tb. N. n = 6. **d** Distribution of microstrain within the defect site in control and loaded tibia on PSD10 and 14. **e** Quantification of the strain in control and loaded tibia. n = 6. PSD = postsurgical day. **P* < 0.05; ***P* < 0.01. Ordinary two-way ANOVA or Student’s t test. Data were mean ± SD.


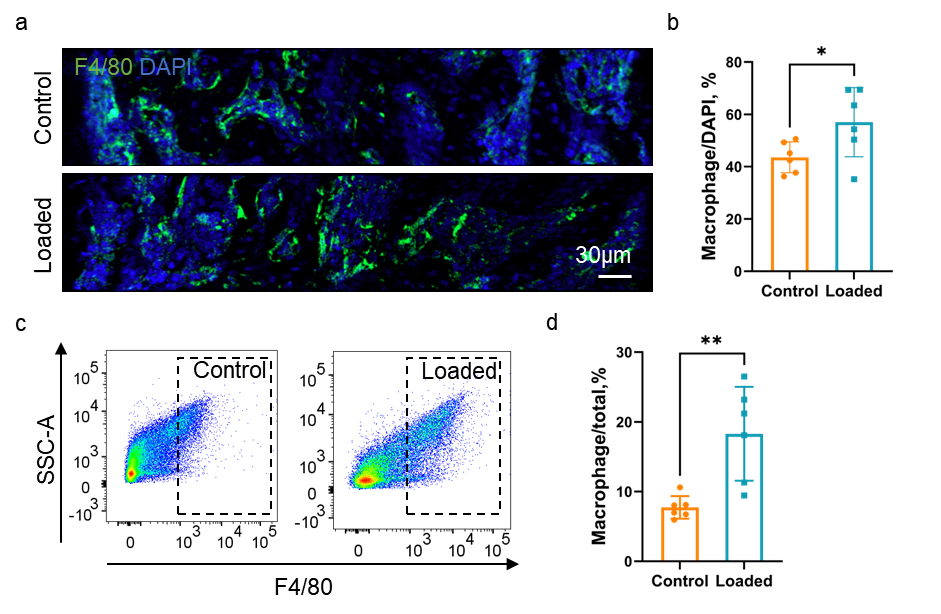


Supplementary Fig. 7 Mechanical loading increased the number of macrophages in bone defect during bone regeneration. **a** Immunofluorescence assay of the expression of F4/80+ macrophages in control and loaded tibia on PSD 10. **b** Quantification of the ratio of F4/80+ macrophages in total cells in response to mechanical loading. n = 6. **c** FACS analysis of F4/80+ macrophages isolated from defect site in in control and loaded tibia. **d** Quantification of the proportion of F4/80+ macrophages in total cells via FACS analysis. n = 6. **P* < 0.05; ***P* < 0.01. Student’s t test. Data were mean ± SD.


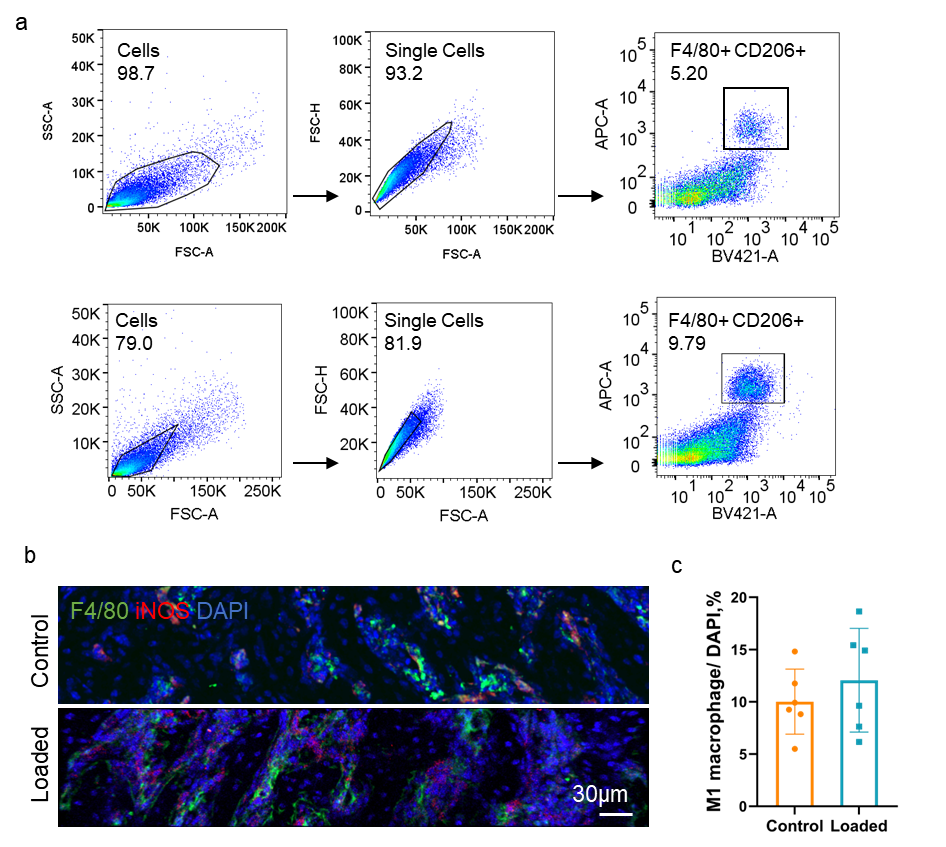


Supplementary Fig. 8 Mechanical loading promoted a minority toward M1 polarization in the bone defect. **a** FACS analysis of F4/80+ CD206+ macrophages (M2 macrophages) isolated from defect site in in control and loaded tibia. **b** Immunofluorescence assay of the expression of F4/80+ iNOS+ macrophages (M1 macrophages) in control and loaded tibia on PSD 10. **c** Quantification of the ratio of M1 macrophages in total cells in response to mechanical loading. n = 6. PSD= postsurgical day. ***P* < 0.01. Student’s t test. Data were mean ± SD.


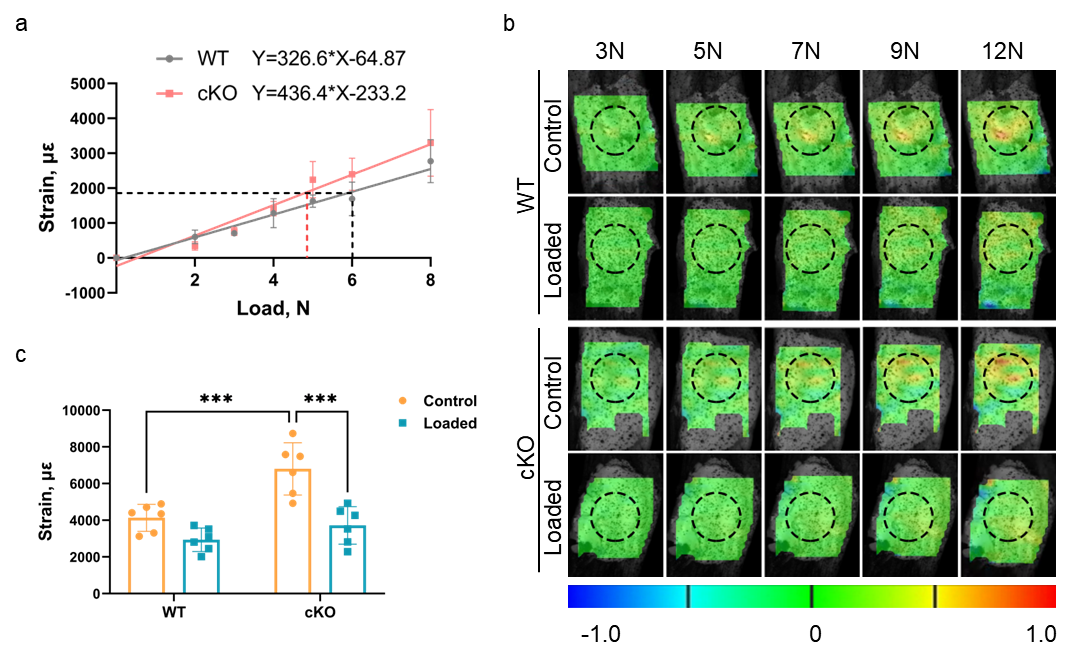


Supplementary Fig. 9 Mechanical loading rescued mechanically-weak new bone tissue caused by periostin knockout. **a** The strain magnitude of the defect site in WT or cKO mice under different loading actions. **b** Distribution of microstrain within the defect site in control and loaded tibia from WT or cKO mice in applied the force of 3, 5, 7 ,9, 12 N. **c** Quantification of the strain in control and loaded tibia. n = 6. ****P* < 0.001. Ordinary two-way ANOVA. Data were mean ± SD.


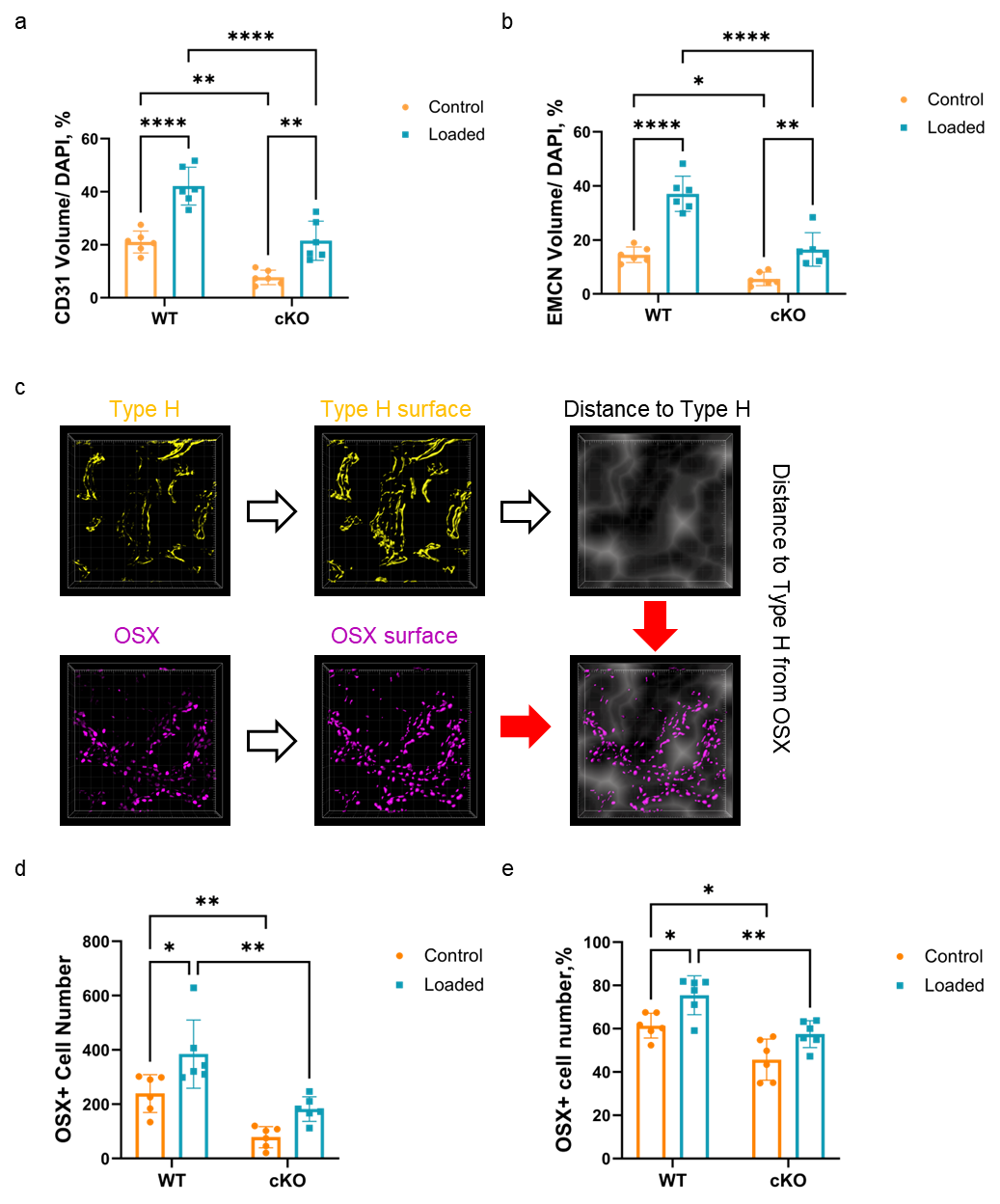


Supplementary Fig. 10 Mechanical loading alleviated low number of OSX+ cells in contact with type H vessels caused by periostin knockout. **a-b** Quantification of the volume of **(a)** CD31+, **(b)** EMCN+ blood vessels in control and loaded tibia. n = 6. **c** Schematic diagram of spatial location analysis between osteoblasts and blood vessels. **d-e** Quantification of the **(d)** number and **(e)** percentage of OSX+ cell numbers in the distance less than 10μm to type H vessels in control and loaded tibia. n = 6. **P* < 0.05; ***P* < 0.01; *****P* < 0.0001. Ordinary two-way ANOVA. Data were mean ± SD.


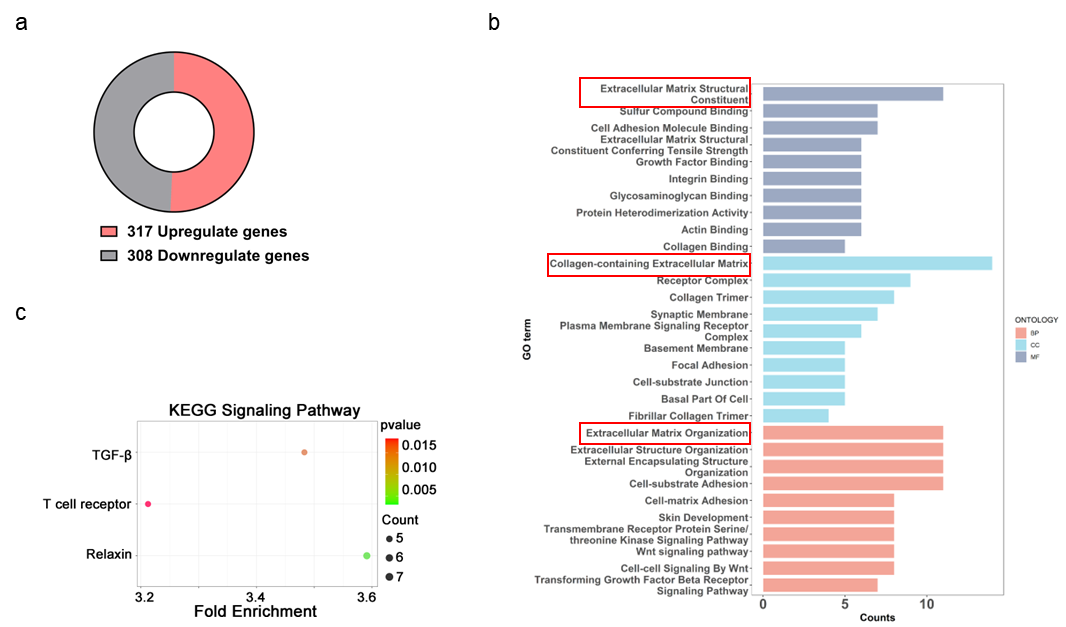


Supplementary Fig. 11 Mechanical loading regulated the bone extracellular matrix in bone regeneration. **a** DEGs (FoldChange > 1.5, p.value < 0.05) from RNA-seq data of the defect area isolated from the control and loaded tibia of WT mice. n = 3. **b** GO analysis of biological processes, cellular component, and molecular function enrichment between control and loaded tibia of WT mice. n = 3. **c** KEGG signaling pathway enrichment analysis of DEGs (FoldChange > 1.5, p.value <0.05). n = 3. **P* < 0.05. Student’s t test. Data were mean ± SD.


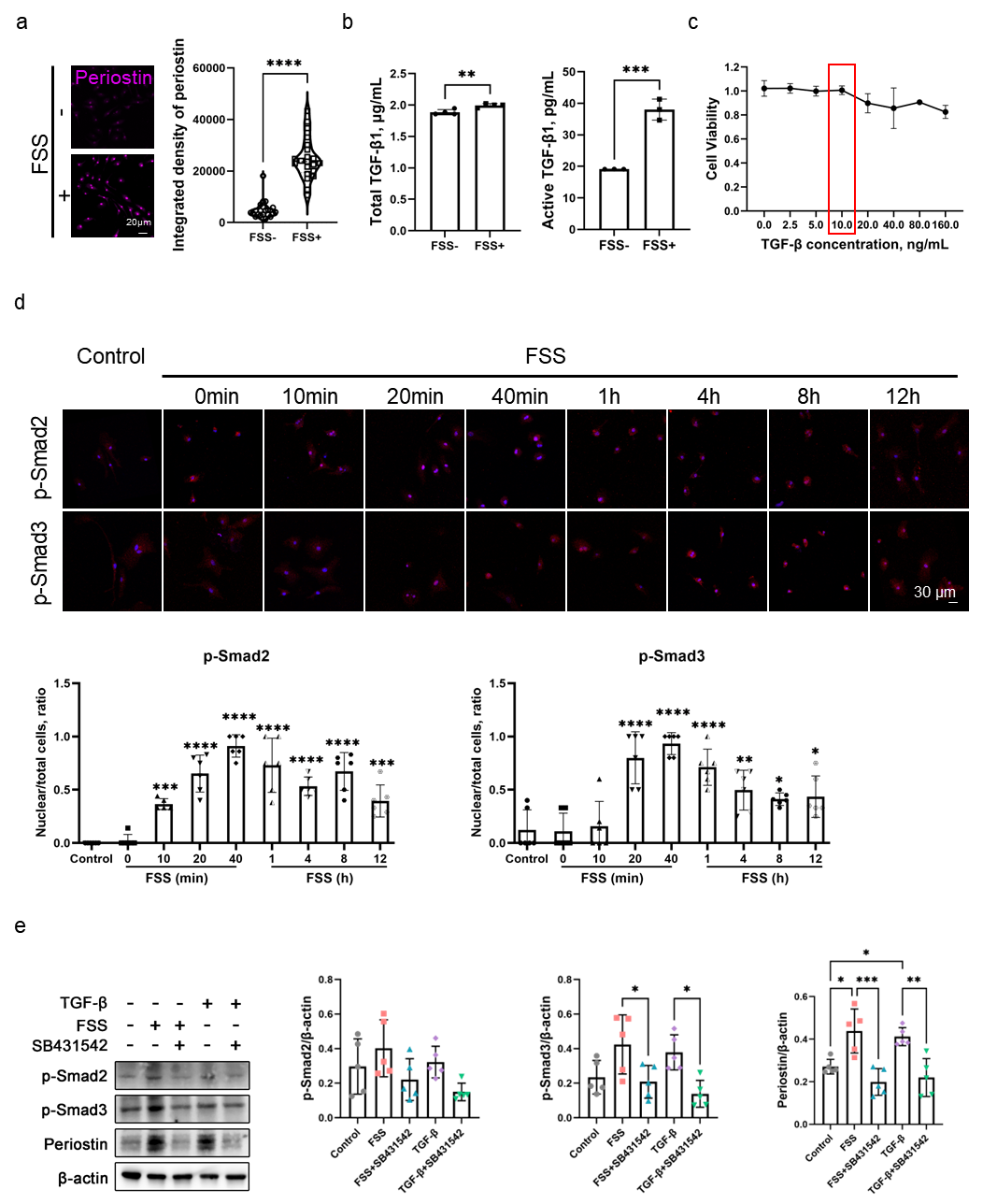


Supplementary Fig. 12 Fluid shear stress increased the expression of periostin in macrophages. **a** Immunofluorescence assay and quantification of the expression of periostin in BMDMs after FSS treatment. n = 20. **b** Expression of total and active TGF-β in BMDMs after FSS treatment by ELISA test. n = 3. **c** Toxicity of TGF-β to BMDMs measured by CCK8. n = 6. **d** Immunofluorescence assay and quantification of the expression of p-Smad2 and p-Smad3 in BMDMs after FSS treatment at timepoints. n = 6. **e** Western blot analysis and quantification of Smad2/3 and p-Smad2/3 expression in SB431542-treated BMDM cells of fluid shear stress and TGF-β treatment. n = 5. **P* < 0.05; ***P* < 0.01; ****P* < 0.001; *****P* < 0.0001. Ordinary one-way ANOVA. Data were mean ± SD.


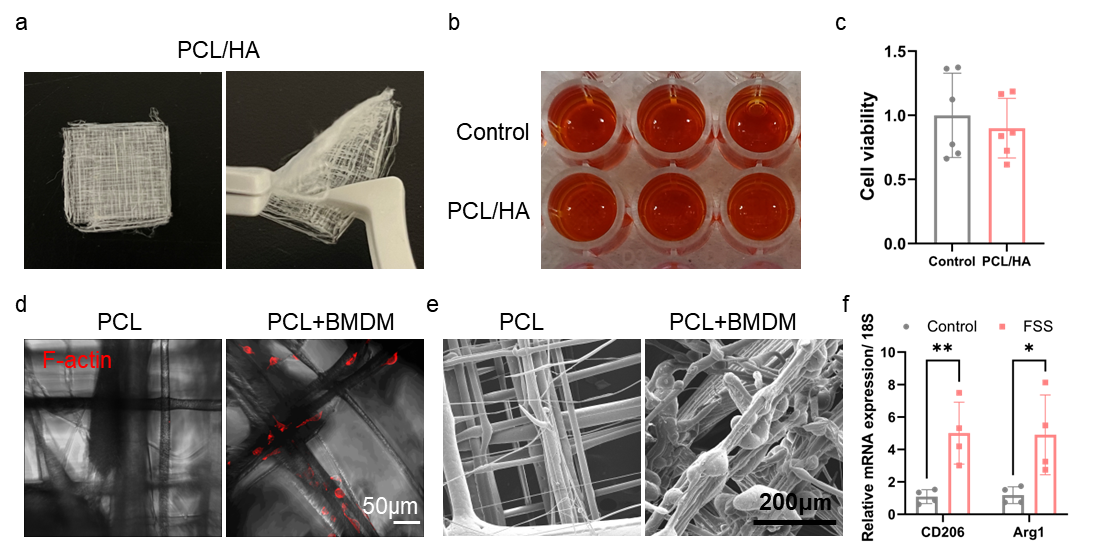


Supplementary Fig. 13 Biocompatibility of PCL/HA membrane. **a** Flexibility of PCL/HA membrane. **b-c** Cytotoxicity of PCL/HA membrane measured by CCK8 test. n = 6. c Toxicity of TGF-β to BMDMs measured by CCK8. n = 6. **d** Immunofluorescence images of the morphology of the PCL/HA with or without BMDMs from C57BL/6J mice. **e** SEM images of the morphology of the PCL/HA with or without BMDMs from C57BL/6J mice. **f** Quantitative RT-PCR analyses of the expression of Arg1, and CD206 in BMDMs after FSS treated. n = 3. **P* < 0.05; ***P* < 0.01. Ordinary one-way ANOVA or Student’s t test. Data were mean ± SD.


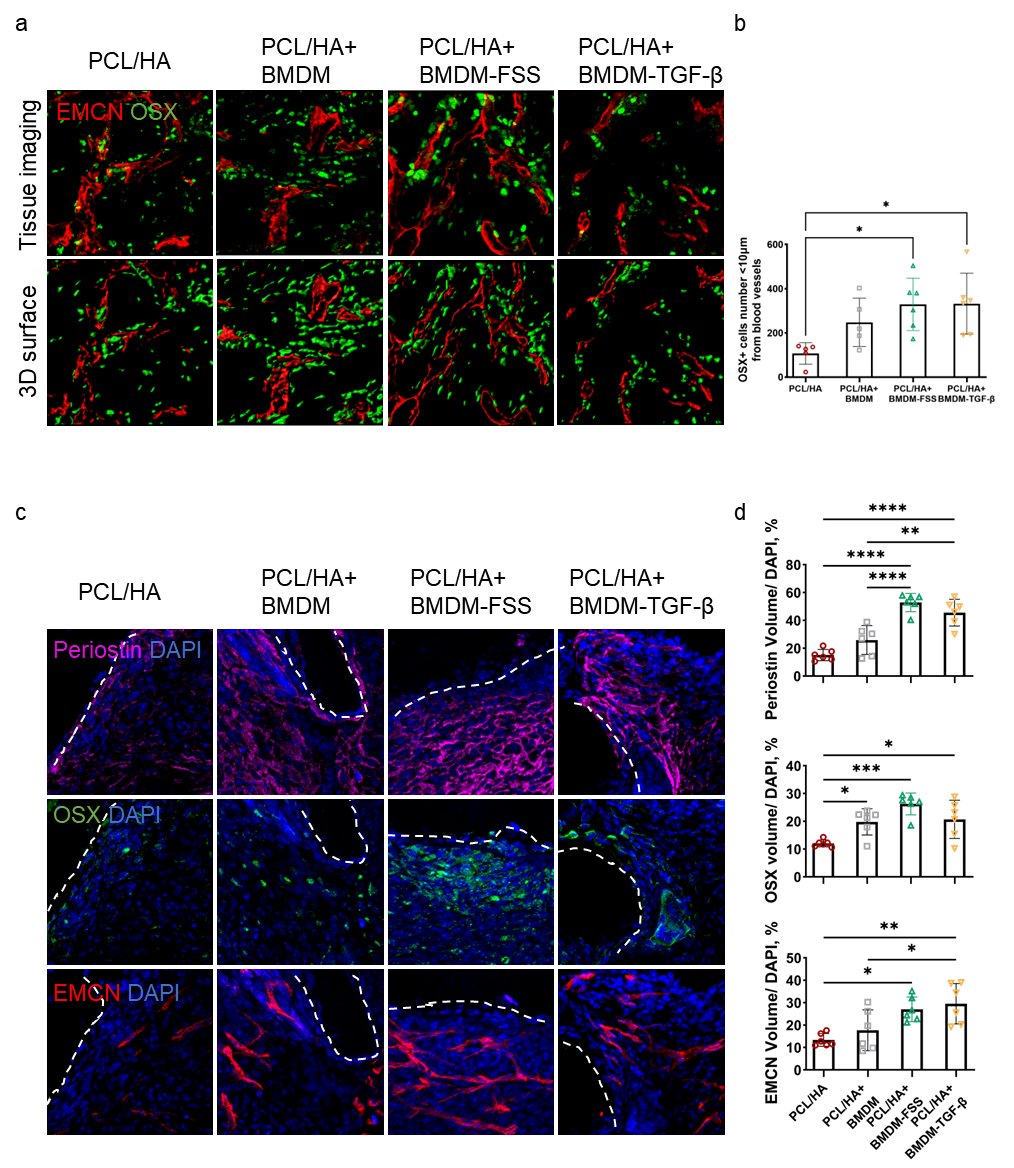


Supplementary Fig. 14 Enhancement of angiogenesis-osteogenesis coupling through mechanically conditioned macrophages. **a** Immunofluorescence assay of the coupling of OSX+ cells and EMCN+ vessels in in PCL/HA, PCL/HA with BMDMs from C57BL/6J mice, PCL/HA with FSS-treated BMDMs, and PCL/HA with TGF-β-treated BMDMs. **b** Quantitative immunofluorescence assay of the number of OSX+ cells in contact with type H vessels. n = 6. **c** Immunofluorescence assay of the volume of EMCN+ vessels, periostin, and the number of OSX+ cells in different implanted subcutaneously groups on PSD 10. **d** Quantitative immunofluorescence assay of the volume of EMCN+ vessels, periostin, and the number of OSX+ cells. n = 6. **P* < 0.05; ***P* < 0.01; ****P* < 0.001; *****P* < 0.0001. Ordinary one-way ANOVA. Data were mean ± SD.

**Supplementary Table 1**

Primers of mouse used for RT-PCR

| Gene | Primers |
| --- | --- |
| F4/80 | Forward 5’- GCATCATGGCATACCTGTTC -3’ |
|  | Reverse 5’- AGTCTGGGAATGGGAGCTAA -3’ |
| iNOS | Forward 5’- TCAGCTACGCCTTCAACACC -3’ |
|  | Reverse 5’- TTCCCAAATGTGCTTGTCACC -3’ |
| CD80 | Forward 5’- TTCGTCTTTCACAAGTGTCTTCA -3’ |
|  | Reverse 5’- TGCCAGTAGATTCGGTCTTCA -3’ |
| CD206 | Forward 5’- CATTCCCTCAGCAAGCGATG -3’ |
|  | Reverse 5’- GGGTTCCATCACTCCACTCA -3’ |
| Arg-1 | Forward 5’- CAGCACTGAGGAAAGCTGGT -3’ |
|  | Reverse 5’- CAGACCGTGGGTTCTTCACA -3’ |
| POSTN | Forward 5’- CCTGCCCTTATATGCTCTGCT -3’ |
|  | Reverse 5’- CCTGCCCTTATATGCTCTGCT -3’ |
| TGF-β | Forward 5’- ATGCTAAAGAGGTCACCCGC -3’ |
|  | Reverse 5’- ATGCTAAAGAGGTCACCCGC-3’ |
| 18s | Forward 5’- GAGAAACGGCTACCACATCC -3’ |
|  | Reverse 5’- CCTCCAATGGATCCTCGTTA -3’ |
