# Supplementary material for: Periostin+ myeloid cells improved long bone regeneration in a mechanosensitive manner
Source: Bone Res. 2024 Oct 15;12:59. doi: 10.1038/s41413-024-00361-5 (PMC11480347; doi:10.1038/s41413-024-00361-5)
Supplement: Supplementary file 2 — Supplementary data - WB [file 41413_2024_361_MOESM2_ESM.pdf]

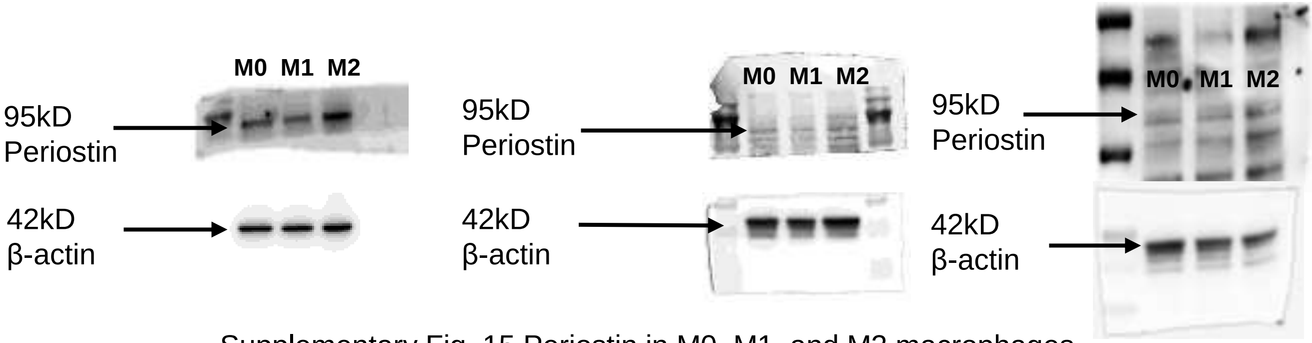

Supplementary Fig. 15 Periostin in M0, M1, and M2 macrophages

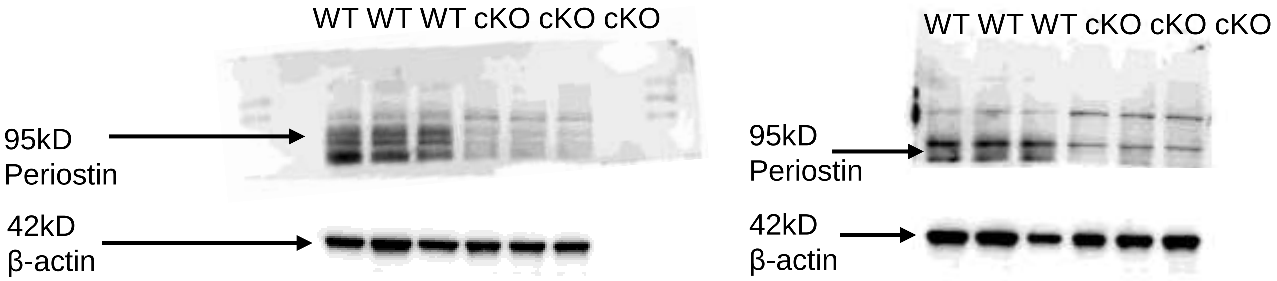

Supplementary Fig. 16 Periostin in BMDMs from WT and cKO mice

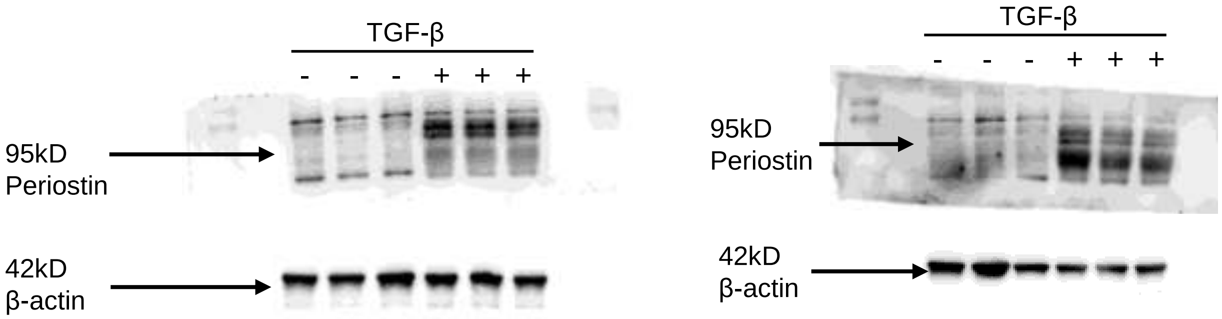

Supplementary Fig. 17 Periostin in BMDMs and TGF-β-treated BMDMs

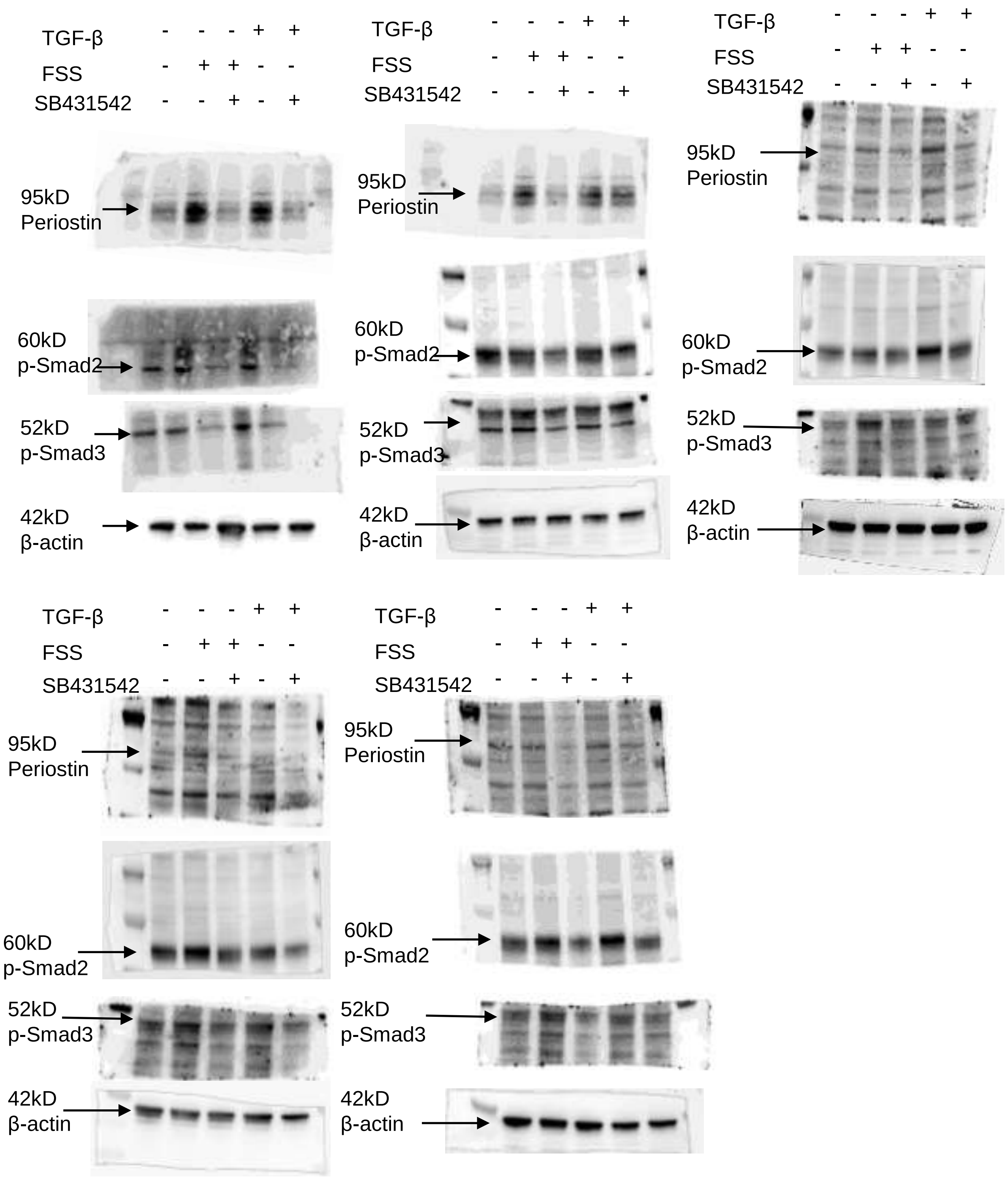

Supplementary Fig. 18 Periostin and p-Smad2/3 in BMDMs stimulated by TGF-β and FSS after SB431542 pretreatment
